# Chronic spontaneous urticaria: update on pathogenesis and therapeutic implications^؆^

**DOI:** 10.1016/j.abd.2025.501198

**Published:** 2025-08-21

**Authors:** Paulo Ricardo Criado, Roberta Fachini Jardim Criado, Hélio Amante Miot, Beatrice Martinez Zugaib Abdalla, Helena Zenedin Marchioro, Renan Rangel Bonamigo

**Affiliations:** a Centro Universitário Faculdade de Medicina do ABC, Santo André, SP, Brazil; b Faculdade de Ciências Médicas de Santos (Fundação Lusíada), Santos, SP, Brazil; c Alergoskin Alergia e Dermatologia, UCARE Center and ADCARE, Santo André, SP, Brazil; d Department of Infectology, Dermatology, Diagnostic Imaging and Radiotherapy, Faculty of Medicine, Universidade Estadual Paulista, Botucatu, SP, Brazil; e Dermatology Residency, Hospital Irmandade Santa Casa de Misericórdia de Curitiba, Curitiba, PR, Brazil; f Service of Dermatology, Santa Casa de Porto Alegre, Porto Alegre, RS, Brazil; g Service of Dermatology, Hospital de Clínicas, Universidade Federal do Rio Grande do Sul, Porto Alegre, RS, Brazil

**Keywords:** Chronic urticarial, Chronic urticaria/therapy, Chronic urticaria/pathology, Immunobiologicals, Small molecules, Urticaria

## Abstract

**Background:**

The understanding of chronic spontaneous urticaria pathogenesis has been increasing recently. The central role of mast cells is being reinforced, but multiple cells, pathways, and mediators are involved in a complex interrelationship. Modern therapies for its management reflect the need to encompass different mechanisms and promise to alter the course of urticaria and the long journey of those with refractory disease. Continuous updating of these aspects is necessary to optimize patient care.

**Objectives:**

To review concepts and advances in the pathogenesis of chronic spontaneous urticaria, in addition to contextualizing promising drug options for its management.

**Method:**

A narrative review was conducted between 1977 and 2024, including relevant articles published in the scientific literature, indexed in the PubMed system.

**Results:**

A total of 25,732 articles were found. Inclusion criteria were determined by the authors' decision regarding their level of importance for furthering knowledge in the areas of pathogenesis and treatment of chronic spontaneous urticaria, with preference given to meta-analyses, systematic reviews, and randomized trials. Regarding therapeutics, 138 articles from the last 15 years were prioritized, in addition to records on ClinicalTrials.gov, and the drugs could be in the clinical trial phase. Immunobiologicals and small molecules hold promise for future treatment regimens for chronic spontaneous urticaria.

**Study limitations:**

Narrative reviews do not provide statistical value to the results and outcomes studied.

**Conclusion:**

A review of the pathogenesis of chronic spontaneous urticaria was conducted, contextualizing these aspects with promising drug options for its treatment, particularly immunobiologicals and small molecules.

## Introduction

Urticaria is a cutaneous inflammatory process of varying intensity in which mast cell degranulation initially leads to vasodilation, increased capillary permeability, and dermal edema (hives), accompanied or not by subcutaneous and/or submucosal edema (angioedema).^1^, ^2^

**Figure 1:**
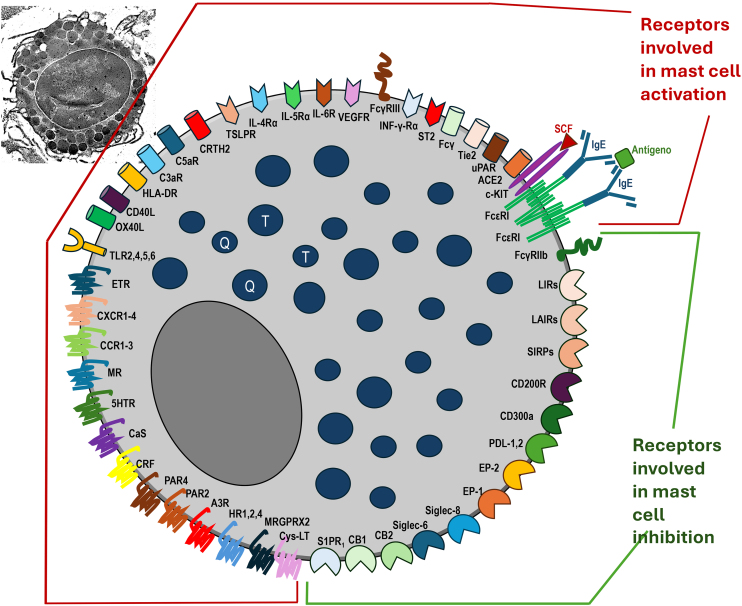
Mast cells and their receptors that stimulate activation, degranulation via IgE-dependent and non-IgE-dependent pathways, synthesis of newly formed mediators, and receptors that suppress mast cell activation. Top left, a still-intact mast cell in the non-urticaria skin of a patient with hives, under transmission electron microscopy (×10,000 magnification). T, Tryptase; C, Chymase.

Mast cell degranulation results from several factors associated with cell receptors, physical stimuli, chemical stimuli, or those linked to immunity (innate and/or adaptive), which promote, within minutes, the release of preformed mediators (stored within intracytoplasmic granules). Newly formed mediators (lipid derivatives and cytokines/chemokines) are released minutes to hours after stimulation of these cells (Fig. 1 and Table 1, Table 2, Table 3).^2^ In general, infections/infestations, foods, and medications are the main causes of acute urticaria.

**Table 1:** Main receptors, ligands and mediators that promote mast cell activation.

Receptors and Type of Mediators	Name	Functions/Actions
Immunoglobulin superfamily receptors	FcɛRI	Expressed on mast cells and basophils as a tetramer composed of subunits (αβγ2), the α subunit responsible for binding the FcɛRI receptor to IgE, the β subunit for regulating receptor expression and signaling, and the γ subunit responsible for signal transduction. FcɛRI circulates free in the blood, either in a soluble form (sFcɛRI) or bound to IgE. IgE binds to FcɛRI with high affinity (Kd ∼10-9 to 10-10), and the dissociation half-life of IgE from FcɛRI is very slow, on the order of weeks. Due to IgE's high affinity for the receptor and slow dissociation, there is long-lasting sensitization of mast cells exposed to specific antigens.
	FcγRIII	When activated by IgG1, in mice, it promotes anaphylaxis
G protein-coupled receptors	Mas-related G protein-coupled receptor X2 (MRGPRX2)	Expressed predominantly in mast cells of the skin, peripheral neurons, basophils, and eosinophils. Activated by eosinophil major basic protein (MBP) and eosinophil cationic protein, fluoroquinolone antibiotics, opioids, neuromuscular blockers, human β-defensin 2 (hBD2), cathelicidin (LL-37), neuropeptides (such as substance P), Calcitonin Gene-Related Peptide (CGRP), Vasoactive Intestinal Substance (VIP), Staphylococcus aureus δ-toxin, hormone receptor modulators, phenothiazines, corticostatin-14, icatibant, cetrorelix, leuprolide, octreotide, sermorelin, atracurium, tubocurarine, rocuronium, and herbal medicines.
	Histamine receptors H1, H2 and H4 (H1R, H2R and H4R)	The binding of histamine to its H1R on mast cells stimulates the release of more histamine and other mediators, increasing the expression of cell adhesion molecules and chemotaxis of eosinophils and neutrophils, in addition to increasing antigen presentation capacity and costimulatory activity in B cells, and suppressing IgE production. Histamine binding to the H2R inhibits mast cell chemotaxis, as well as that of eosinophils and neutrophils, suppressing Th2 responses. Stimulation of the H4R leads to increased intracellular calcium influx, mast cell degranulation, and the release of cytokines, cysteinyl leukotrienes, and leukotriene B4 (LTB4). It is also relevant to the histaminergic pathways of cutaneous sensory nerve endings that conduct pruritus.
	Adenosine receptor (A3R)	Adenosine receptors differ in the type of G protein they recruit, their effect on adenylyl cyclase (AC) activity, and the downstream signaling pathway they trigger. Adenosine can both increase and inhibit mast cell degranulation, indicating that its effects on these receptors are controversial and still need to be clarified. Depending on the study model, A1, A2b, and A3 receptors demonstrate anti- or pro-inflammatory activity.
	Cystil-Leukotriene Receptors	Cysteinyl leukotrienes (CysLTs), released from mast cells, are important mediators of allergy. Type 1 receptors for CysLTs (CysLT1Rs) are involved in accelerating IgE-mediated MC activation.
	Protease-activated receptor (PAR) types 2 (PAR2) and 4 (PAR4)	PAR2: can be activated by proteases (chymase, tryptase)
		PAR4: activated by thrombin.
	Corticotropin-releasing factor (CRF) receptor	CRF1 receptor: induces the release of VEGF. They are co-receptors of VEGFR-2.
	Calcium sensor (CaS)	Increases mast cell activity.
	Serotonin receptor (5-HTR)	Amplifies mast cell activation
	Acetylcholine receptor (Muscarinic M)	Its binding to acetylcholine evokes mast cell degranulation.
	Chemokine receptors: CCR 1-3 and CXCR1-4	The CCR3 receptor has high affinity for eotaxin-1/CCL-11, eotaxin-2/CCL-24, and eotaxin-3/CCl-26 and mediates mast cell migration
	Endothelin receptor (ET_A,B_)	Via this receptor, endothelin-1 determines mast cell degranulation and can induce the production of TNF-α and IL-6.
Activated complement receptors	C3aR	Activates mast cells, amplifying degranulation
	C5aR	Activates mast cells, amplifying degranulation
Maturation, differentiation, and activation receptor (type II receptor of tyrosine kinase)	c-Kit (CD117)	Its ligand targets Stem Cell Factor (SCF), for which it has high affinity (Kd −200 pM), causing receptor dimerization and phosphorylation of tyrosine residues. It is expressed in mast cells, hematopoietic and non-hematopoietic cells (such as melanocytes and interstitial cells of Cajal), and some tumors. In mast cells, it is expressed from their progenitors until their differentiation and maturation, and their development and survival depend on its activation by SCF, mediated intracellularly by the PI3K pathway, causing degranulation in response to allergens and cytokine production.
Neuropilin Receptors	Receptors NRP 1 and 2	Relevant in angiogenesis
Tyrosine kinase receptors for angiopoietin	Tie 1 and Tie 2	Its ligand is angiopoietin, which, together with VEGFs, is important for the proliferation, migration and survival of endothelial cells, including the formation of lymphatic vessels.
Neurokinin Receptor	NK1	This receptor is expressed when the mast cell is stimulated by IL4 or SCF.
Pattern Recognition Molecular Receptors (PAMPs and DAMPs)	TLR 1,2, 3,4, 5, 6,7, 8,9 e 10	Recognition of bacteria and fungi in particular, as well as peptides such as Gram-negative lipopolysaccharides (LPS), which can reach the bloodstream due to dysbiosis of the intestinal microbiome. Viral receptors (TLRs 3, 5, 7, and 9) are intracytoplasmic.
Major histocompatibility antigen	MHCII	HLA-DR
Sphingosine-1phosphate receptors	S1PR_2_	Upregulates both allergen-induced degranulation and chemokine secretion
Glycosylphosphatidylinositol (GPI)-anchored cell surface receptor	CD48	Expressed on mast cells, eosinophils, and nearly all hematopoietic cells, including basophils, it is regulated by bacterial and viral products and immune-associated proteins. It is important as a costimulatory molecule in lymphocyte activation, facilitating cell adhesion and innate responses to bacteria (such as S. aureus and its exotoxins). Its soluble form (sCD48) is elevated in lymphoproliferative diseases, Sjögren's syndrome, and asthma compared to healthy individuals.
Receptors of Interleukins and Other Cytokines	IL-33	ST2 receptor: its ligand, IL-33, is a member of the IL-1 superfamily, initiating and amplifying the responses of type (2) innate lymphoid cells by stimulating the synthesis of IL-5 and IL-13 by these cells. IL-33 is also synthesized by mast cells after IgE-mediated activation and acts autocrinely on the mast cells themselves, stimulating them. IL-33 produced by the bronchial epithelium stimulates mast cells to produce IL-1β and IL-6, inducing the differentiation of Th0 lymphocytes into the Th17 phenotype after challenge with ovalbumin (OVA).
	IL-4	IL-4 Rα (type I) and IL-4Rα coupled to IL-13Rα2 (type II): when IL-4 binds to these receptors, it decreases mast cell proliferation, increases the expression of ICAM-1 (Intercellular Adhesion Molecule-1) and reduces the expression of c-Kit, but in synergy with SCF, IL-4 promotes mast cell proliferation and directs the production of cytokines in the IgE-dependent pattern.
	IL-5	IL-5Rα: activates mast cells, but is crucial for stimulating eosinophils, prolonging their survival, activation, adhesion to endothelial cells, differentiation and maturation, in addition to promoting the interaction between mast cells and eosinophils
	IL-6	IL6R: activated by IL-6, also synthesized by mast cells and innate immunity
	Vascular endothelial growth factor (VEGF) types 1 and 2	VEGF1R and VEGF2R: Mast cells can express receptors for VEGF, which they produce.
	Interferon gamma receptor	INFγ-Rα: INFgamma can determine mast cell apoptosis.
	Thymic Stromal Lymphopoietin (TSLP)	TSLPR: is a heterodimer composed of the α-chain and α-chain of IL-7R, and is the target of TSLP, produced primarily by epithelial and endothelial cells, which causes differentiation and proliferation of mast cells. It also induces the production of chemokines and the synthesis of Th2-type cytokines.
Prostaglandin D2 Receptor	CRTH2 or DP2	PGD2 is the main metabolite derived from arachidonic acid, released by IgE-activated mast cells. CRTH2 or DP2 is present intracellularly in mast cells and on the membranes of other cells such as LTh2, eosinophils, basophil macrophages, and dendritic cells. By binding to the PGD2 receptor, it mediates cell chemotaxis and promotes degranulation of mast cells, basophils, and eosinophils.
Costimulatory molecules	CD40L (CD154)	Transmembrane glycoprotein that binds CD40 on other immune cells, such as CD4+ T cells and platelets. The interaction between CD40L and CD40 on B cells is relevant for immunoglobulin class switching and memory B cell generation. Blocking the CD40L-CD40 interaction reduces the generation of regulatory T cells (Tregs). Activation of CD40 allows the expression of costimulatory molecules such as CD80 and CD86.
	OX40L (CD134L)	Involved in interaction with antigen-presenting cells (macrophages). Interacts with OX40 (CD134) of T lymphocytes, including promoting Th17-dependent inflammation, together with TNF-α and IL-6.
		The interaction between OX40L and its ligand OX40 on T cells induces T cell expansion and proliferation and decreases the immunosuppressive effect of regulatory T cells (Treg)
Angiotensin-converting enzyme type 2 receptor	ACE2	Studies demonstrate its presence in mast cells of the respiratory tree.

**Table 2:** Main receptors, ligands and mediators that suppress mast cell activation.

Receptors and Type of Mediators	Name	Functions/Actions
Cannabinoids	CB1	Under the action of endocannabinoids (anandamide and 2-arachidonoylglycerol), it promotes regulatory actions. In mast cells, it has anti-inflammatory effects, with CB1 suppressing mast cell degranulation by increasing cytosolic cAMP levels.
	CB2	Under the action of endocannabinoids (anandamide and 2-arachidonoylglycerol), it promotes regulatory actions. In mast cells, it has anti-inflammatory effects by suppressing the release of pro-inflammatory mediators.
Sphingosine-1phosphate receptors	S1PR_1_	Its activation by agonists decreases allergic inflammation in the airways and reduces the accumulation of eosinophils and T cells
Immunoglobulins	FcγRIIb (low-affinity receptor for IgG type b) (expressed only in mice, but not in human mast cells)	In murine mast cells, FcγRIIb can cross-link adjacent FcɛRI on the cell membrane with the same multivalent ligand and form an immune complex and inhibit FcɛRI-dependent mast cell activation, preventing degranulation and synthesis of newly formed mediators.
CD300 Family	CD300a	Its ligands are phosphatidylserine and phosphatidylethanolamine (expressed in the early stages of apoptosis/cell death) as membrane signals for phagocytosis. In the intracellular space, its action activates the SHP-1 protein, dephosphorylates SYK, and decreases intracellular calcium influx. CD300a expression on mast cells can be inhibited by eosinophil major basic protein (MBP) and eosinophil-derived neurotoxins.
Siglecs Family (sialic acid-binding immunoglobulin-like lectins)	Siglec-6	Its activation prevents mast cell activation.
	Siglec-7	Its activation results in inhibition of mediator release via FcɛRI-dependent pathway, by cross-linking with adjacent FcɛRI on the cell membrane.
	Siglec-8	Stimulated by IL-5, IL-33, or GM-CSF, mast cell apoptosis is induced. Activation of mast cell mediators results in FcɛRI-dependent inhibition of mast cell mediator release.
Leukocyte Ig-like receptor (LIR)	Inhibitory receptors LIR-1, LIR-2 and LIR-3	LIR-1 is expressed in mature mast cells and LIR2 and LIR-3 in mast cell progenitors. They promote inhibition of mast cell activity.
Membrane glycoprotein receptor belonging to the SIRP family	SIRP-α	Its activation inhibits FcɛRI-mediated mast cell activation in mice
CD200R	CD200R	Its activation inhibits FcɛRI-mediated mast cell activation
c-lectin mast cell function-associated antigen (a variant of lectin-like type II transmembranal glycoprotein)	MAFA	Inhibits degranulation and cytokine release by IgE-activated mast cells in rat mucosa
Platelet-Endothelial Cell Adhesion Molecule-1	PECAM-1	Its activation inhibits FcɛRI-mediated mast cell activation
Leukocyte-associated I-like receptor (LAIR)	LAIR-1	Its activation results in the reduction of preformed mediators and the release of cytokines. Its ligand is collagen.
Prostanoid-E Receptors	EP2 e EP4	Prostaglandin E2 (PGE2) binds to the EP2 receptor and inhibits mast cell degranulation, cytokine transcription, and eicosanoid production via stimulation of FcɛRI. Binding to EP4 inhibits mast cell activation. One of the mechanisms of urticaria caused by nonsteroidal anti-inflammatory drugs is the decrease in PGE2 synthesis and loss of its mast cell-silencing mechanisms.
Programmed death receptor	PDL1	Induces mast cell apoptosis

**Table 3:** Major preformed and newly formed receptors, ligands and mediators.

Receptors and Type of Mediators		Name	Functions/Actions
Preformed (generally stored in granules of 0.2 to 0.8 μm in diameter, of skin mast cells. TC mast cells, chymase-tryptase positive)	Amines	Histamine	Vasodilation, expression of VCAM in the vascular endothelium activating endothelial cells, increased capillary permeability, stimulation of pruritus
		Polyamines	Vasodilation
	Proteoglycans	Heparin	Anticoagulation
		Chondroitin sulfate	Inhibits the activation of other connective tissue mast cells
		Serglycin	Proteoglycan to which heparin binds and whose function is to maintain the homeostasis of mast cell granules to be secreted.
	Proteases	Tryptases (expressed only in mast cells)	Tryptase-α, Tryptase-βI Tryptase-βII, Tryptase-βIII, Tryptase-γ, Tryptase-δ: Increased capillary permeability, stimulation of pruritus via PAR2
		Chymase-1 (expressed only in skin mast cells)	Protease stored in skin mast cell granules, promoting inflammation and tissue destruction
		Cathepsin G	
		Carboxypeptidase A	
		Granzyme B 3	
	Lysosomal enzymes	β-glucoronidase	Glycosidase that participates in the catabolism of mucopolysaccharides
		β-Hexosaminidase	Involved in hydrolysis mechanisms.
		Arylsulfatase	Catalyzes reactions of sulfonated hexoses.
	Cytokines	TNF-α	Stimulates TH1 polarization
		bFGF	Stimulation of fibroblasts in the regeneration of the intercellular matrix
		IL-4	Stimulates type 2 inflammation
		SCF	Maturation, differentiation and increased survival of mast cells
		VEGFb	Increased capillary permeability and angiogenesis
Neoformed (synthesized minutes to hours after mast cell stimulation)	Lipids	Prostaglandin D2 (PGD2)	Vasodilation and increased capillary permeability
			Recruitment and activation of Th2 lymphocytes
			Chemotaxis and activation of basophils and eosinophils
			Migration of type 2 innate immune lymphoid cells (ILC2)
		Leukotriene B4 (LTB4)	Chemotaxis of neutrophils, mast cells, and dendritic cells
			Recruitment of CD8+ lymphocytes
		Leukotriene C4 (LTC4)	Eosinophil migration
		Leukotriene D4 (LTD4)	Migration of eosinophils and neutrophils
		Leukotriene E4 (LTD4)	Migration of eosinophils and ILC2
		Prostaglandin E2 (PGE2)	Stabilization of mast cells and their decreased activation and migration of dendritic cells
		Platelet activating factor (PAF)	Vasodilation, increased capillary permeability, increases adhesion of blood cells to the vascular endothelium and promotes migration to the extravascular space
	Cytokines	Interleukin-1 (IL-1) (increased serum expression in CSU)	Recruitment of eosinophils, neutrophils, migration of dendritic cells, activation of the inflammasome; IL-1β determines plasma extravasation from vessels
		Interferon type I (INF-α and INF-β) and II (INF-γ and has decreased serum expression in CSU)	Secreted in viral infections such as respiratory syncytial virus. It leads to the aggregation of lymphocytes, eosinophils, mast cells, macrophages, and neutrophils.
		IL-2 (increased serum expression in CSU)	Proliferation of effector T lymphocytes and B lymphocytes; development of Treg cells; growth factor for B lymphocytes and stimulation of antibody synthesis
		IL-3 (increased expression in CSU lesions)	Activation of eosinophils and basophils; enhancement of FcεRI expression in basophils and stimulation of their longer survival
		IL-4 (increased serum expression in CSU)	Activation of T lymphocytes and basophils; increased humoral immunity; recruitment of eosinophils; differentiation from Th0 to Th2 with induction of type 2 inflammatory response and monocytes; differentiation into M2 macrophages; factor of increased survival for T and B lymphocytes; promotes immunoglobulin class switching, determining the production of IgE and IgG1 by B lymphocytes.
		IL-5 (increased serum expression in CSU)	Increase in the number of eosinophils due to increased adhesion and chemotaxis capacity to the skin.
		IL-6 (increased serum expression in CSU)	Differentiation of B lymphocytes and subsequent production of IgG, IgM and IgA; increased mast cell proliferation, maturation and reactivity (priming)
		IL-8 (increased serum expression in CSU)	Acts as a chemoattractant for T lymphocytes, basophils, eosinophils, NK cells, and neutrophils
		IL-9 (increased serum expression in CSU)	Acts as a growth factor for mast cells and T lymphocytes; inhibits Th1-type cytokines and promotes the proliferation of CD8+ lymphocytes and mast cells
		IL-10 (increased serum expression in CSU)	Inhibits Th1 and Tc1 cell function; activates B lymphocytes and induces autoantibody production by B cells
		IL-12	Recruitment of CD8+ T lymphocytes; induction of Th1 lymphocyte responses
		IL-13 (increased serum expression in CSU)	Activation of mast cells and eosinophils; increased eosinophil survival
		IL-16	T lymphocyte recruitment and dendritic cell migration
		IL-17A (increased serum expression in CSU)	Induction of pro-inflammatory cytokines and chemokines, with recruitment of neutrophils
		IL-18	Dendritic cell migration
		IL-25 (increased expression in CSU lesions)	Induction of Th2 responses and inhibition of Th1 and Th17 responses; induction of the production of IgE and IgG1, IL-4, IL-5, IL-9 and IL-13
		IL-31 (increased serum expression in CSU)	Induction of IL-6 and IL-8, CXCL1, CXCL8, CCL2 and CCL8 in eosinophils
		IL-33 (increased serum expression in CSU)	Increases allergic stimulation of mast cells and basophils; induces IL-31 production in mast cells; increases integrin expression in basophils and eosinophils, leading them to the skin; promotes mast cell maturation
		TNF-α ( “de novo” synthesis: increased serum expression in CSU)	Promotes activation of endothelial cells, proliferation of effector T lymphocytes, increases the expression of ICAM-1 in eosinophils allowing them to migrate to the skin; activation of monocytes and macrophages, recruitment of neutrophils and proliferation of T lymphocytes
		GM-CSF	Determines migration of dendritic cells to lymph nodes
	Chemokines	CCL1	Recruitment of T cells and monocytes
		CCL2	Recruitment of T cells, monocytes, and neutrophil migration
		CCL3	Recruitment of T cells and monocytes
		CCL4	Recruitment of T cells and monocytes
		CCL5	Recruitment of CD8+ T cells, monocytes, basophils, eosinophils, NK cells and dendritic cells
		CCL7	Recruitment of T cells: monocytes, basophils, eosinophils, NK cells, immature dendritic cells, and hematopoietic progenitor cells
		CCL18	Recruitment of naive T cells, CD4+ and CD8+ T lymphocytes, memory T lymphocytes, B lymphocytes, and immature dendritic cells
		CCL20	Recruitment of B lymphocytes, memory effector T lymphocytes, recruitment of CD11b+ dendritic cells
		CXCL1	Neutrophil recruitment
		CXCL2	Recruitment of neutrophils, basophils, and eosinophils
		CXCL3	Neutrophil recruitment
		CXCL8	Recruitment of neutrophils and CD4+ T lymphocytes
		XCL1	Dendritic cell migration and antigen cross-presentation
		CXCCL1	Recruitment of monocytes and T lymphocytes and increased survival

The main mediator responsible for the initial events of urticaria is histamine, which binds to H1 and H2 receptors on the vascular endothelium, determining the effects of vasodilation, increased capillary permeability (in conjunction with other mediators released by mast cells), and pruritus, its cardinal symptom, initially caused by stimulation of histaminergic cutaneous sensory pathways (via H1 and H4 receptors) and the action of tryptase.^2^

Hives are transitory, generally lasting in the same location for no more than 24 hours, while angioedema is more long-lasting (varying between 48 and 96 hours).^1^ Urticaria is classified according to its temporal evolution as acute (<6 weeks) or chronic (>6 weeks). Although hives are common in Chronic Urticaria (CU), 43% to 59% of patients also present with angioedema; however, approximately 10% present with angioedema alone (mast cell angioedema).^1^

Over time, in addition to pre-synthesized substances (histamine, tryptase, and other proteases) released by damaged mast cell granules, “de novo” synthesized mediators, such as lipid derivatives (prostaglandins and leukotrienes) and cytokines/chemokines, are released by mast cells, activating the cutaneous vascular endothelium (expression of cell adhesion molecules such as PECAM-1/CD31, E-selectin, P-selectin, ICAM-1, VEGF expression, and tissue factor production), and leading to the chemotaxis of other blood cells to the skin, such as basophils, lymphocytes, eosinophils, and neutrophils. These recruited cells, like a feedback loop, determine the establishment of chronic and intermittent inflammatory responses in distinct areas of the skin, which are recurrent and of varying intensity. Other mediators, besides histamine, play a key role in the chronicity of the disease.^2^

Conceptually, Chronic Urticaria (CU) is classified as induced: related to identifiable and generally external triggering stimuli, such as cold, heat, pressure, water, solar radiation, or even cholinergic or symptomatic dermographism; or spontaneous, when the trigger is not always the same, appearing in different situations throughout the disease's history.^1^

This article aims to present a review of the pathogenesis of Chronic Spontaneous Urticaria (CSU), as well as to support the rationale for its current and developing therapies.

## Methods

This narrative review addressed the literature from January 1977 to October 2024, in PubMed/Medline, using the terms (*idiopathic* OR *spontaneous* = 4.169) AND *urticaria, prevalence* AND *urticaria* (3.320), *genetic* AND *urticaria* (2.486), *autoantibodies* AND *urticaria* (766), *coagulation* AND *urticaria* (757), *gut* AND *microbiota* AND *urticaria* (43), *stress* AND *urticaria* (482)*, treatment* AND *urticaria* (13.709), totaling 25.732 articles. The authors selected relevant articles on these topics that have provided advances in these areas. Priority was given to articles and records in Clinical Trials (ClinicalTrials.gov) addressing aspects of the pathogenesis of chronic spontaneous urticaria, its currently recommended treatment, and the prospects for new medications such as small molecules or biological agents.

## Prevalence and Natural History of Urticaria

Different population groups have variable prevalence rates for CU. Latin America has the highest prevalence, 1.5% (95% CI 0.0–6.0), followed by Asia, 1.4% (95% CI 0.5–2.9), Europe, 0.5% (95% CI 0.2–1.0), and North America, 0.1% (95% CI 0.1–1.0). Women are more affected by CU than adult men, while no gender differences were observed in children.^3^

In Brazil, a retrospective study estimated a 1.7% prevalence of CU among dermatological patients.^4^ Another study, which analyzed retrospective data from 2011, 2012, and 2015, from a national health and well-being survey with 36,000 respondents, resulted in a 0.41% prevalence of CU.^5^ In Brazil, two studies demonstrated a higher incidence of CU in adults among women, with estimates ranging from 80%^4^ to 86%.^6^

There is little published data on the natural history of urticaria, and some of it has methodological limitations, most focusing on the duration of CSU.^7^ In general, the frequency of progression of acute urticaria (AU) cases to CU is not adequately explored, and some studies are conducted in hospital settings rather than population-based.^7^

In an evaluation of data from the Korean national healthcare system between 2002 and 2013, with a population sample of 1,025,340 individuals, a total of 4.8% were identified with urticaria, which determined its annual incidence rate ranging from 45.7 to 82.5 per 100,000 person-years.^7^ In this study, among all patients with urticaria; 6.1% were identified with progression to CU. Among these; 46.6% already had CU when they were first diagnosed with urticaria. The remainder had AU at their first diagnosis, but they subsequently developed CU when the disease recurred after a median of 614 days.^7^ Over the five-year and ten-year periods, the cumulative incidence rates of CU among all patients with urticaria ranged between 6.3% and 7.8%, respectively, and approximately 50% of patients with CU experienced remission after 11 months of disease, with CU remission rates ranging from 52.6% in the first year of disease, 78% in the third year, and 89% at 5 years.^7^

Higher financial income was associated with higher CU remission rates.^7^ Furthermore, the study demonstrated that several other clinical factors interfered with the progression to CU, conferring a higher risk: age ≥ 10 years, male gender, urban residence, and autoimmune thyroid disease. Women were more affected by AU than men in the age range between 20–44 years and 45–64 years, which may be linked to estrogen, which stimulates humoral immunity.^7^

Regarding the average duration of CSU, in patients aged ≥12 years, it is estimated to last approximately five years, although it can persist for longer periods in severe cases.^8^ In a review that included eight observational studies and two reviews, aimed at determining the duration of the CSU course and disease remission rates, the average age ranged from 34 to 68 years, and the proportion of women ranged from 61% to 80%.^8^ The proportion of patients who achieved CSU remission within the first year of disease ranged from 21% to 47%, while remission rates reported in the fifth year of disease were between 34% and 45%.^8^ Based on a four-week consecutive CSU remission, cumulative remission rates ranged from 9% to 38% in the first year, from 29% to 71% in the fifth year of disease, and from 52% to 93% in the twentieth year of CSU.^8^

Chronic pruritus without hives or angioedema occurred in 12.2% of patients. There was a greater likelihood of CSU persisting in patients diagnosed with hypothyroidism (HR = 0.43; 95% CI 0.29–0.60) and with each point increase in the UAS7 severity score (HR = 0.93; 95% CI 0.92–0.95).^9^

Thus, regarding the natural course of the disease, urticaria is a condition that will affect approximately 10% to 20% of the world's population at some point in their lives. There is a consensus that acute spontaneous urticaria is common; however, it is often associated with upper respiratory tract infections. Most of these infections are self-limited, and less than 10% progress to CU, although some studies indicate higher rates.^7^, ^10^, ^11^ CU, in turn, persists for periods of > 1 year in most patients, significantly impairing quality of life, causing psychiatric and systemic comorbidities, and incurring high costs to the healthcare system.^7^, ^8^, ^10^, ^11^

## Genetic and Epigenetic Basis

CU is a multifactorial disease, in which the hypersensitivity reaction is based on a genetic and epigenetic basis that inflicts susceptibility.

Regarding histocompatibility antigens, HLA-Bw35 has been associated with CU and other endocrine diseases in the Japanese population.^12^ CD4+ T lymphocytes in the infiltrate, as well as vascular endothelium, sweat glands, and nerves, express HLA-DR.^13^, ^14^, ^15^

In Caucasian patients with CSU, a strong association with HLA-DRB1*04 (HLA-DR4) was found in patients who manifested histamine-releasing activity in their serum, demonstrating that, despite being heterogeneous, CU has an autoimmune pathogenesis in a subgroup of patients.^16^

In a sample of Chinese individuals, CSU patients had lower methylation in 439 genes, with 86.5% of these genes hypomethylated, especially on chromosome 6, suggesting an autoimmune behavior similar to diseases such as rheumatoid arthritis, multiple sclerosis, and lupus. The study also identified that the sphingolipid pathway, particularly sphingosine-1-phosphate (S1p), may be involved in CSU, as hypomethylation increases its production, influencing inflammation and the immune response. Indeed, the prevalence of autoimmune diseases in CSU patients is high, and many have specific antibodies and a family history of autoimmunity.^17^, ^18^

Under homeostatic conditions, S1p levels are low in tissues, but mast cells can release S1p in response to stimuli, which increases capillary permeability and recruits immune cells, contributing to allergic inflammation. S1p, through the s1p1 receptor, promotes Th17 T-cell differentiation and can polarize lymphocytes toward the Th2 immunophenotype, in addition to suppressing the differentiation of thymic and extrathymic T-regulatory (LTreg) cells, leading to chronic inflammation.^19^ The autocrine effect of s1p on sip2 receptors on mast cells themselves leads to degranulation.^19^

In a GWAS study, CSU was associated with SNPs in the genes TDGF1, HLA-G, PTPN22, LILRA3, and IGHG1/IGHG2, which explain 8.07% of CSU heritability at the estimated disease prevalence point of 0.5%–1% in the general population. The first three are associated with CSU autoimmune phenotypes.^20^ These alterations underlie macrophage activation in the pathogenesis of CSU.^20^, ^21^

The TDGF1 gene increases the phagocytic activity of macrophages and stimulates the production of pro-inflammatory and anti-inflammatory cytokines through the NF-Kb pathway.^20^, ^22^ The transcription factor c-Maf determines the production of IL-10 by type 2 phenotype macrophages (M2 macrophages), helping to perpetuate inflammation mediated by Th2 lymphocytes, which maintain the inflammatory process and contribute to the chronicity of urticaria.^20^, ^21^ The HLA-G gene identified among the alleles of SNPs related to CSU encodes HLA-G, an HLA class I molecule that induces immunotolerance by suppressing the functions of NK cells, CD4+ and CD8+ T cells, and dendritic cells. Therefore, elevated levels of soluble HLA-G are observed in patients with allergic rhinitis and asthma, which correlate with serum IgE specific to allergens.^20^, ^23^

Two studies demonstrated an association between CSU and autoimmune diseases with the CRTH2^24^ and ORAI1 genes.^25^ Other studies indicated a relationship between CSU and the HLA-DRB1*04,^26^ HLA-DRB1*01, HLA-B44 and HLA-DRB*15 alleles,^27^ TGF-β1,^28^ PTPN22 (a factor that regulates the production of anti-TPO and anti-TG IgG)^29^ and IL-2.^30^ In particular, the presence of an association between CSU and the CRTH2 and TGF-β1 genes is related to cases associated with allergic/atopic asthma, and with HLA-DRB1*01, HLA-B44 and HLA-DRB*15 with autoimmune diseases, but not atopic disease.^20^

In a study with knocked-down Human Mast Cell Line-1 (HMC-1) after incubation with thrombin to mimic the pathogenesis of CSU, Fang et al.^31^ observed the relevance of the CCL2 (Chemokine C-C motif ligand-2) and CH25H (Cholesterol 25-Hydroxylase) genes and the tumor necrosis factor signaling pathway.

In another GWAS study, two loci associated with CSU risk were identified: HLA-DQA1 and ITPKB. Inositol 1,4,5-Triphosphate Kinase B (ITPKB) is part of a family of kinases that phosphorylate inositol as a second messenger in the calcium signaling pathway.^32^ The regulation of inositol-1,4,5 is involved in neuronal signaling and immune function, including mast cell and basophil degranulation, suggesting that ITPKB may play a role in the loss of tolerance in CSU and enhance calcium-dependent secretion responses in mast cells.^32^

**Figure 2:**
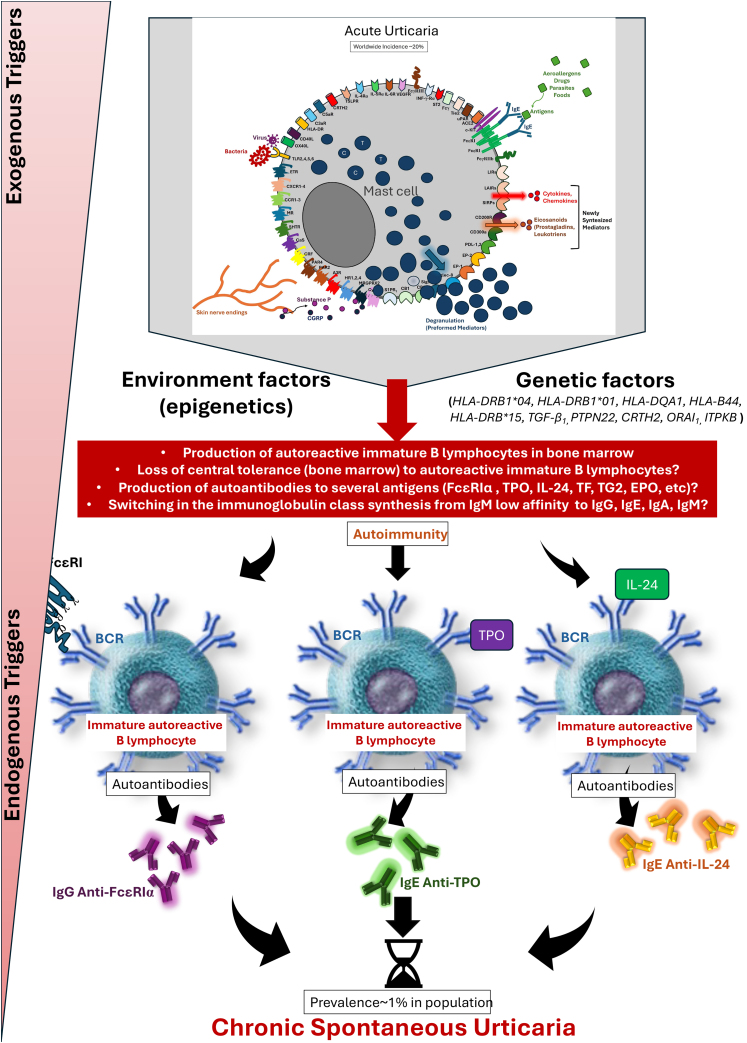
Main epigenetic and genetic mechanisms involved in acute urticaria in its evolution to chronic spontaneous urticaria.

Based on these models, stimulated primarily by exogenous factors, a small proportion of patients with acute urticaria, under the influence of endogenous factors (such as genetic and epigenetic aspects), interact within the phenotypic spectrum of CSU, where different HLAs and gene polymorphisms determine a breakdown in immune tolerance, with a predisposition to the disease and an association with other autoimmune conditions, which together determine the background of the disease pathogenesis (Fig. 2).

## Pathogenesis

**Figure 3:**
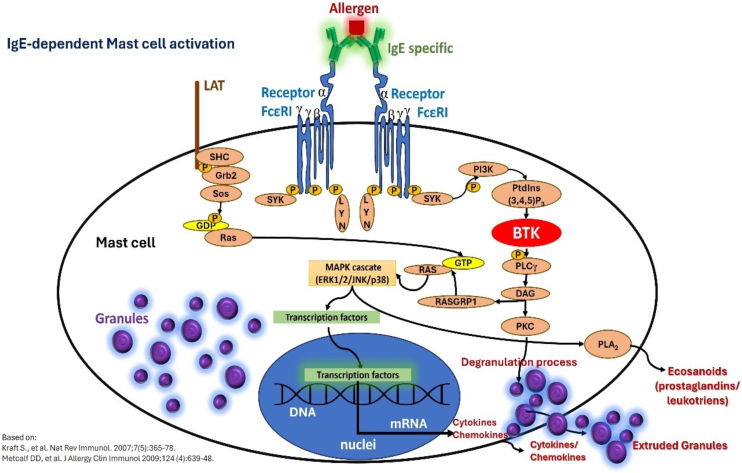
Mast cell activation and degranulation to external antigens in IgE-mediated reactions outside the context of chronic spontaneous urticaria: IgE specific to a given allergen, binding to contiguous FcεRI receptors on the mast cell membrane, and intracellular signaling events mediated by Bruton's tyrosine kinase (BTK). The phenomenon culminates in mast cell degranulation and synthesis of newly formed mediators.

**Figure 4:**
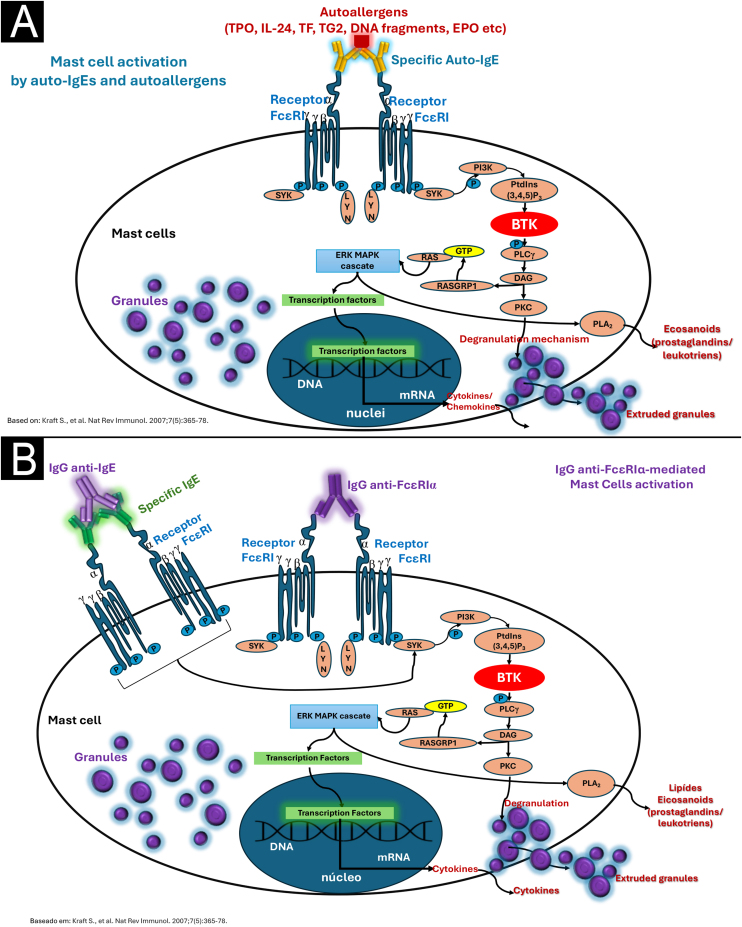
(A) Type I autoimmunity mechanism (autoallergy), with the participation of IgE alloantibodies against circulating autoantigens. (B) Mechanism IIb (autoimmunity) mediated by the presence of IgG autoantibodies, possibly IgM and IgA, against the alpha subunit of the high-affinity IgE receptor (FcɛRIα) on mast cells and basophils, or against the patient's own IgE, either circulating or bound to FcɛRI receptors. In both mechanisms, mast cell degranulation and synthesis of newly formed mediators occur through the activation of specific intracellular and nuclear signaling pathways, which share the activation of Bruton's tyrosine kinase (BTK).

The pathogenesis of CSU is centered on the activity of mast cells, whose degranulation in the skin and subcutaneous/submucosal tissues leads to the formation of hives and angioedema, and its cardinal symptom, pruritus. Therefore, CSU is considered a mast cell-dependent disease, with the participation of basophils and other cell elements that are attracted to the skin, involving various immune mechanisms such as autoimmunity, inflammation, coagulation factors, and activation of mast cell membrane receptors (Figure 3, Figure 4), which cause chronic inflammation.^10^, ^32^

Several authors classify CSU as an autoimmune disease, due to genetic findings and the coexistence of other autoimmune diseases.^32^

Malmros H.^33^ in 1946 described the first finding of what is termed an “autoreactivity” phenomenon in CU patients, when he injected the own serum (autologous serum) of six CU patients, as in an intradermal test, and observed the formation of erythema and hives within 30 minutes of the injection, suggesting passive transfer of antibodies. Subsequently, in 1986, it was demonstrated that seven of 12 tests of autologous serum from patients with Chronic Idiopathic Urticaria (CIU) reinjected intradermally, produced erythema and hives at the injection site.^33^, ^34^, ^35^

In recent decades, considerable discoveries have been revealed about the mechanisms involved in mast cell activation, with the description of new activating and inhibiting receptors expressed on their cell membrane surface.^36^ Most mast cell progenitors already express the c-Kit receptor and FcɛRI on their membrane, which are essential for the survival and activity of these cells.^36^ Different cytokines, chemokines and adhesion molecules, such as IL-4, IL-5, IL-6 IL-15, TNF-α, CCL-2 and vascular cell adhesion molecule (VCAM-1) released into the tissue microenvironment regulate the expansion, homing and maturation of mast cell precursors. However, the binding of Stem-Cell Factor (SCF) to the c-Kit receptor remains the most potent signal of mast cell differentiation, proliferation, and survival.^32^ Different authors have described the presence of IgG and IgM autoantibodies directed against IgE and/or its membrane receptor alpha subunit (FcɛRIα),^37^, ^38^, ^39^, ^40^ in addition to basopenia in the peripheral blood of these patients.

Subsequent advances led to the understanding of the existence of two main autoimmune mechanisms that determine the so-called “autoreactivity” in patients with CSU: type I autoimmunity (or autoallergic, aaCSU), primarily mediated by IgE specific to self-antigens (endogenous peptides),^41^ with IgE directed against serum thyroperoxidase, and type IIb autoimmunity (autoimmune CSU, aiCSU), mediated by IgG,^38^ and to a lesser extent also by IgM and IgA, directed against IgE or the FcɛRIα receptor, expressed on mast cells and basophils.^32^

Both mechanisms (endotypes) suggest the common principle that CSU is fundamentally an autoimmune disease, not influenced by exogenous agents/factors.^42^ However, the observation that autoantibodies are not always detected in patients with CSU indicates that mechanisms other than autoimmunity, whether immunological or non-immunological, are involved in the pathogenesis, contributing to the understanding of different CSU phenotypes.^32^

Type I/aaUCE is inherent in the existence of IgE antibodies to several currently recognized antigens such as thyroperoxidase or thyroid peroxidase (TPO), thyroglobulin (TG), tissue factor (TF), eosinophil peroxidase (EPO), double-stranded DNA (dsDNA), eosinophil cationic protein (ECP), interleukin-24 (IL-24), and IgE anti-FcɛRI.^11^, ^42^, ^43^ The most common autoantigens are TPO and IL-24, and IgE-antiTPO and IgE-antiIL-24 demonstrate *in vitro* the activation of mast cells and/or basophils.^42^, ^44^, ^45^ IgE anti-IL-24 is present in approximately 70% to 80% of patients with CSU, and its serum concentration in patients with CSU correlates with disease activity.^44^

When the patient's autologous serum (autohemotherapy) was used to treat CSU, the Autologous Serum Test (ASST) was negative in 28% and 34% of patients, respectively, at weeks 9 and 21. However, this did not correlate with the overall response to treatment, as there was a reduction of slightly more than 50% in the antihistamine doses required for CSU treatment compared to the period prior to autohemotherapy.^46^ The selective activation of mast cells in the skin by IgE autoantibodies can be explained by the occurrence of certain autoallergens, expressed almost exclusively in the skin, such as IL-24, and cross-reactivity with certain proteins, such as TPO (absent in the skin) and EPO (expressed in the skin).^42^

Type IIb/ai CSU occurs in the form of IgG anti-FcɛRIα in approximately 20%–50% of patients.^11^, ^47^ These autoantibodies activate mast cells and basophils, for example via IgE and FcɛRI, but are present in 8% of patients with CSU when strict criteria are applied, which include the presence of triple positivity in the following tests: ASST, immunoassay for IgG autoantibodies, and Basophil Activation Test (BAT).^47^ Type IIb autoimmune CSU is characterized by the presence of high disease activity (elevated UAS7), concomitant with other autoimmune diseases (such as Hashimoto's thyroiditis), low serum levels of total IgE, high levels of IgG anti-thyroid peroxidase (TPO) antibodies, basopenia (< 10 cells/μL) and eosinopenia (< 50 cells/μL),^48^ in the peripheral blood and poor therapeutic response to antihistamines, poor response or partial response to omalizumab, in addition to a good therapeutic response to cyclosporine.^47^ In mechanism IIb, the presence of IgG (subtypes IgG1 and IgG3) was demonstrated in 24% of the patients evaluated, and more than half of the patients had IgM (60%) or IgA (5%) against the FcɛRI receptor (most common), when these antibodies were tested by the ELISA method and against IgE.^42^, ^49^ In clinical practice, it is recommended to evaluate the relationship between serum levels of anti-thyroid peroxidase IgG/total serum IgE, which in these patients can result in values above 15, as an assessment of the existence of aiUCE (type IIb), since serum levels of total IgE <40 IU/mL are frequently observed.^50^

In fact, many patients with CSU show the coexistence of auto-IgE and IgG autoantibodies (coexistence of type I and type IIb mechanisms), which indicates an overlap between these two endotypes.^43^, ^51^ In Brazil, the authors observe 38% of patients with type I mechanism endotypes, 51% with concomitant type I and IIb mechanism endotypes, 9% with IIb endotypes, and 2% of patients who did not meet criteria for any endotype.^51^ Thus, overlapping autoreactivity mechanisms in CSU types I and IIb, with IgG and IgE anti-TPO, can coexist in the same patient. However, patients with aiUCE with or without coexisting aaUCE are generally women, with higher levels of antiperoxidase autoantibodies (both IgG and IgE anti-TPO), and demonstrate a greater negative impact on quality of life, while patients with aaUCE without association with aiUCE are younger individuals.^36^, ^52^ However, considering different studies, less than 35% of patients with CSU will not have any autoantibodies (non-autoimmune endotype).^51^, ^52^

In addition to autoantibodies, another factor involved in the amplification of mast cell degranulation is the activation of the complement system through the alternative pathway. This is particularly relevant in the mechanism of type IIb CSU, where after activation of FcɛRI on the mast cell membrane by IgG-anti-FcɛRI, C5a (anaphylatoxin) is generated and this acts as a ligand for the C5aR receptor expressed only on cutaneous mast cells, and exacerbates mast cell degranulation, worsening and amplifying CSU, which makes the skin the target organ in CSU due to the unique expression of C5aR, sensitizing it to inflammatory and autoimmune processes.^36^

Understanding the pathogenesis of CSU has allowed us to clarify the action of therapeutic agents, such as the monoclonal antibody omalizumab, directed against human IgE, sequestering it and suppressing the expression of its membrane receptors (FcɛRI) on mast cells and basophils, so that there is a decrease in the density of antigen to which the anti-FcɛRI IgG is directed, with a consequent decrease in the signaling of mast cell and basophil degranulation.^53^ Consequently, cross-linking between two contiguous FcɛRI receptors on the cell membrane, a necessary step for degranulation, is reduced.^53^ Furthermore, as complement activation via the alternative pathway, which is caused by the aggregation of IgG1 and IgG3 to the FcɛRI antigen, responsible for complement activation, with the use of omalizumab, the expression of FcɛRI on the cell membrane of mast cells was reduced. This reduces the interaction between IgG-anti-FcɛRI and the receptor antigen, consequently decreasing the generation of C5a and its binding to the receptors C5aR on the membrane of cutaneous mast cells, silencing the signals that potentiate the activation of these cells, CSU protagonists.^53^

In addition to the FcɛRI receptor, mast cells can be activated by other activating receptors on their surface, including the Mas-related G protein-coupled receptor X2 (MRGPRX2), the C5Ra receptor, protease activated receptors (PAR1 and PAR2, which have trypsin, tryptase, thrombin, and the FVIIa/FXa/TF complex as ligands), a molecule homologous to chemoattraction receptors expressed on Th2 cells (CRTh2), and cytokine receptors (ST2, for IL-33; IL-4Rα receptor for IL-4, among others such as IL-5, IL-6, IL-15, IL-25, thymic stromal lymphopoietin [TSLP], and TNF-α), and the c-Kit receptor.^36^

The diversity of mast cell receptors (FcɛRI, MRGPRX2, PAR1 and PAR2, CRTh2, c-Kit [CD117], and receptors for various cytokines and histamine H1 and H4) makes this cell capable of being subjected to a stimulus that influences an underlying response (priming).^11^ Activation of FcɛRI involves several intracytoplasmic signaling proteins, such as LYN, splenic tyrosine kinase (SYK), and Bruton's tyrosine kinase (BTK), which promote subsequent phosphorylation signals in other proteins and induce mast cell activation and degranulation.^11^ Thus, the first step of FcɛRI-mediated signaling is the phosphorylation of the FcɛRI beta chain (FcɛRIβ-chain) and gamma chain (FcɛRIγ-chain) by the LYN protein, followed by the activation of SYK and BTK.^11^ Thus, cytosolic BTK is the central positive regulator of mast cell activation and cytokine production in FcɛRI-mediated stimuli.^11^ In addition to its role in mast cell activation, BTK is required for the B Cell Receptor (BCR) signaling pathway and subsequent antibody synthesis.^11^

In addition to the membrane receptors that activate mast cells, these cells have receptors that inhibit their function, such as CD200R, CD300a, FcγRIIb, and sialic acid-binding immunoglobulin-like lectin 8 (Siglec 8), which can block their activation and interaction with other ligands.^11^

Of growing interest is the MRGPRX2 receptor, a G-protein-coupled receptor with seven transmembrane domains, highly expressed in mast cells of patients with CSU and overregulated in severe forms of the disease, participating in degranulation mechanisms called pseudoallergic/neurogenic.^36^ Its activation occurs by several mediators, including compound 48/80, eosinophil-derived mediators (major basic protein, MBP, and EPO), neuropeptides (such as substance P, vasoactive intestinal peptide, VIP), innate host defense peptides (catechecidin), small molecule drugs (nicotine antagonists and neuromuscular blocking drugs), opioids, antibiotics (vancomycin, ciprofloxacin, levofloxacin, and moxifloxacin), and iodinated contrast media, all of which are involved in the development and exacerbation of CSU. After activation of MRGPRX2, there is an increase in calcium in the CM cytosol, mediating Ca2+ channels, Gai, Gaq, extracellular signal regulated by kinase, PI3K/AKT, and phosphoinositide phospholipase Cγ with subsequent degranulation and further cytokine release.^36^ Communication between eosinophils and mast cells, independent of IgE-related mechanisms, can occur in CSU due to MBP and EPO binding to MRGPRX2 receptors, contributing to the maintenance of CSU lesions.^36^

Cutaneous mast cells chronically exposed to IL-33 (alarmin) have their FcɛRI-mediated degranulation attenuated; however, IL-33 can sensitize them to greater acute reactivity to MRGPRX2 receptor ligands.^36^ IL-33's half-life is reduced by the action of proteases and histamine released during degranulation, which constitutes a negative feedback loop, controlling its actions.^36^

### Inflammation and coagulation in CSU

Some patients with CSU demonstrate features of chronic inflammation, including increased mast cell numbers, increased eosinophils, basophils, and other cell elements infiltrating the dermis, increased cytokine expression, neovascularization, and increased expression of vascular adhesion molecules.^10^ However, several other cell types also participate in the pathogenesis, which can be inferred from resistance to several mast cell-targeted medications.^42^ The higher number of mast cells in the skin of patients with CSU is associated with more active urticaria, so that patients with active CSU also have a higher number of mast cells in the skin, compared to patients with CSU in remission and healthy controls.^54^

Histopathologically, more than 90% of patients with CSU have a non-necrotizing perivascular cell infiltrate with no evidence of immunoglobulin or complement deposition, with a prominent increase in mononuclear cells accompanied by a ninefold increase in the number of mast cells with signs of degranulation. The authors speculated that CSU would be an “allergic reaction” -type of disease, even without an identifiable antigen. Furthermore, the infiltration of mononuclear cells and eosinophils in the perivascular environment of the dermis would suggest a molecular immunopathology in CSU similar to late-phase allergic reactions.^55^ Also in this study, the authors identified that the perivascular infiltrate in hives consisted of 47% T lymphocytes and 22% monocytes.^55^

There are different cytokines, cell adhesion molecules, chemokines and enzymes increased in the blood of patients with CU (CSU, symptomatic dermographism, cold urticaria, heat urticaria, cholinergic and delayed pressure urticaria), compared to healthy controls, such as: TGF-β1, IL-2, IL-4, IL-5, IL-6, IL-10, IL12p70, IL-13, IL-17, IL-18, IL-18 BP, IL-21, IL-23, IL-31, INF-γ, TNF-α, TNF-β, CCL5/RANTES, sICAM-1, sVCAM-1 and Transglutaminase-2 (TG2) activity).^54^

Serum levels of IL-33 and TSLP were evaluated in 50 patients with CSU and 38 healthy controls. Elevated serum levels of these alarmins were observed in the patients, including a correlation between IL-33 levels and UAS7 and DLQI scores, concluding that IL-33 may be a diagnostic and prognostic biomarker in CSU.^56^, ^57^

In addition to the increased number of mast cells (CCL3+) in the hives of patients with CSU, a perivascular infiltrate of CD4+/CCR5+ lymphocytes, monocytes, neutrophils, eosinophils, basophils, and macrophages (mostly of the M2 phenotype) was observed, contributing to amplifying the inflammatory state of the disease.^21^, ^36^, ^42^, ^54^^,^^55^, ^58^ In this context, cytokines detected in the skin with hives (lesional skin) indicate response patterns. Inflammatory Immune System type 1 (INFγ) and type 2 (IL-4, IL-5); type 2 inflammation is maintained by the production of alarmins (IL-25, IL-33, TSLP), which are mediators of the innate immune system, especially involved in allergic responses.^36^, ^56^

Originally, the presence of autoreactive CD4+ T cells in the blood of patients with CSU, specifically targeting the FcɛRiα antigen, generated the first evidence of the sequence of autoimmune events in CSU, as in other autoimmune diseases: its onset is related to the activation of autoreactive T cells, stimulated by INFγ (type 1 inflammatory reaction, with initial participation of Th1 CD4+ T cells). Autoreactive T cells (in response to INFγ) are detected earlier than autoantibodies in CSU patients, suggesting that both are present at distinct stages of the CSU course.^59^, ^60^ In parallel, the IL-23/IL-17 and TNF-α axis may contribute to the maintenance of inflammation in CSU.^36^

There is an increase in Th17 cells and IL-17A expression in both CD4+ T cells and mast cells in the skin of patients with severe CSU, in hives, and in apparently normal skin, where these cells are arranged in close proximity, supporting the involvement of T cells in the pathogenesis of CSU.^60^ This observation led to the hypothesis that pro-inflammatory cytokines such as IL-17 and microparticles derived from T lymphocytes can induce mast cell degranulation, in a transient and short-lived manner, which can resolve spontaneously. Once activated, CD4+ cells and mast cells are placed close to each other, and a new exacerbation of urticaria occurs, which is one of the explanations for the episodic/transient nature of the appearance of erythema and hives in CSU.^60^

Thus, the cells that infiltrate the skin in CSU migrate from the blood to the cutaneous compartment in response to different chemotactic factors, for example eotaxin, MCP3, RANTES, IL-5, C3a, C5a, TNF, IL-17 and Platelet Activating Factor (PAF), released by mast cells, activated endothelial cells (by histamine and TNF-α, initially, and also by thrombin, IL-25, IL-33, VEGF), LTh2, dermal fibroblasts and other cells.^11^ Cell adhesion molecules, such as P-selectin, E-selectin, ICAM, VCAM and PECAM, are highly expressed in the hives (lesional skin) of patients with CSU due to the action of histamine, tryptase, and other factors.^11^ This explains why 10% to 15% of patients with CSU present with eosinopenia and basopenia in their peripheral blood, which occurs due to the migration of these cells from the blood to the skin. This is associated with CSU activity, the presence of autoantibodies, and a poor therapeutic response to antihistamines and omalizumab.^11^

**Figure 5:**
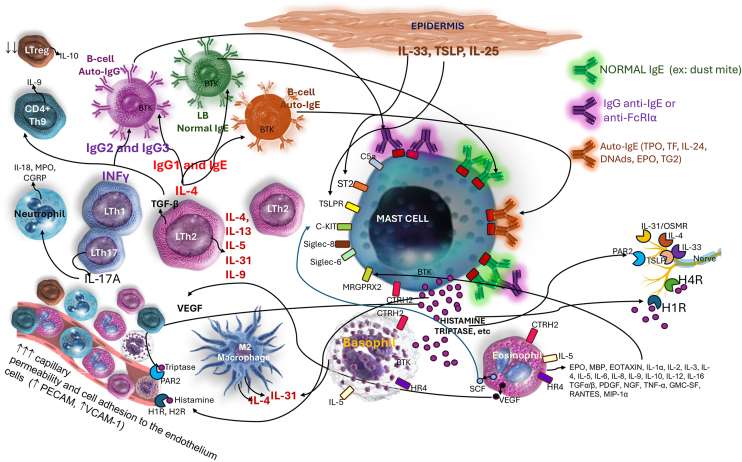
Main inflammatory interactions between mast cells and different cell elements in chronic spontaneous urticaria.

In the early stages of hives formation, 30 minutes after injection of autologous serum from CSU patients, neutrophils and eosinophils are observed in the perivascular area of the dermis (along with T lymphocytes). They increase in number within two hours and decrease around 48 hours (neutrophils) or later (eosinophils and lymphocytes).^11^ Communication between mast cells and eosinophils is relevant in CSU, since both activate each other, with eosinophils releasing SCF, stimulating mast cells, and these producing IL-5, PAF, TNF and eotaxin (Fig. 5).^11^

### Cutaneous activation of the coagulation cascade and complement

Eosinophils and endothelial cells of the dermal microvasculature can express tissue factor (TF) on their surfaces, resulting from various stimuli, such as histamine, VEGF, lipopolysaccharide (LPS), TNF, IL-6, IL-33, and IL-1β, promoting coagulation activation.^11^

Blood coagulation has two main pathways: extrinsic and intrinsic. In the pathogenesis of CSU, the extrinsic pathway triggered by TF is relevant in many patients. Elevated plasma levels of Prothrombin Fragment 1+2 (PF1+2), Fibrin/Fibrinogen Degradation Products (FDP), and D-dimers rose in the blood in proportion to the activity and severity of CSU symptoms.^61^, ^62^, ^63^, ^64^ When small amounts of FVIIa bind to TF on the surface of eosinophils and activated monocytes in the cellular infiltrate of hives and to activated endothelium in the dermal microvasculature, the extrinsic coagulation pathway is activated in the transudate plasma, along with Phosphatidylserine (PS) and Ca2+, and subsequently activates factor Xa, which converts prothrombin (FII) to FIIa (thrombin), which generates fibrin (Fia) from fibrinogen (FI) and, as a waste product, PF1+2.^65^ Plasmin from the fibrinolytic pathway of coagulation then degrades fibrin into PDF and D-dimers.^65^ Fibrin can stimulate Toll-Like Receptor 4 (TRL4), including on mast cells.^65^

Although activation of the coagulation cascade occurs in CSU, it is notable that it occurs as a local process, with active fibrinolysis and no risk of thromboembolic events.^65^, ^66^ TF expression in CSU hives is thus due to activated eosinophils expressing TF on their cell membranes and due to TF expressed by dermal endothelial cells in response to the synergy of two classes of its inducers, Group 1 (LPS, TNF-α, IL-33, IL-1β, which activate the nuclear factor kappa B-related signaling pathway, NF-κB) and Group 2 (histamine and VEGF, which activate the intracellular pathway linked to phospholipase C).^65^ TF expression in endothelial cells can be suppressed by adenosine (a metabolite of ATP).^65^

As subsequent events, there is both activation of PAR1,2,3,4 receptors by activated factors of the extrinsic coagulation pathway [FVIIa activates PAR_2_, FXa activates PAR_1,2,3_ and prothrombin (IIa or FIIa) activates PAR_1,3,4_] which act on different target cells such as mast cells (where PAR_2_ is intensely expressed in urticaria lesions), stimulating the production and release of inflammatory mediators, mitogenesis and in the vascular endothelium promoting capillary hyperpermeability, proliferation, chemotaxis and cell differentiation.^65^ Activated coagulation factors of the extrinsic pathway (FXa, FIIa and plasmin) also act by triggering the alternative complement pathway, converting C3 into the C3b+C3a complex, which, acting on C5, generates C5a and C5b, which act as anaphylatoxins, with C3a and C5a binding to their receptors (C3aR and C5aR) on mast cells and basophils and amplifying the stimulation of these cells.^65^

### Intestinal microbiota and chronic urticaria

CSU occurs with intestinal dysbiosis, and its relevance in the pathogenesis of the disease has been highlighted, presumably by promoting signals that reduce the activation threshold of mast cells (such as IL-33 and bacterial lipopolysaccharides (LPS)) or stimulate inhibitory signals (short-chain fatty acids (SCFAs) such as butyrate and propionate) in these cells.^67^

LPS influence the activation thresholds of mast cells or promote their priming and are produced by the intestinal microbiota.^67^ SCFAs promote epithelial barrier integrity and function by stabilizing Hypoxia-Inducible Factor (HIF), stimulating the expression of tight junction molecules [occludin-1, Mucin 2 (MUC-2), and Zonula Occludens-1 (ZO-1)], and producing antimicrobial peptides. This protects enterocytes and prevents LPS from reaching the bloodstream, which can reach the skin and bind to TLR4 on mast cells, activating them and producing inflammatory mediators such as TNF-α and IL-13.^67^ SCFAs act by stimulating IL-10, which has anti-inflammatory properties, and inhibiting mast cell activation after stimulation by IgE, and also without IgE participation via epigenetic regulation.^67^

SCFAs are responsible, in part, for promoting intestinal homeostasis and its immune balance, being produced and released by Firmicutes, including *Clostridium leptum* and *Roseburia* spp., which regulate the growth and virulence of enteric pathogens, such as *Escherichia coli* and *Klebsiella*, LPS producers.^67^ SCFAs such as butyrate and propionate can facilitate the differentiation of regulatory T lymphocytes (Tregs), which control Th2 (CD4+) LT cells and their cytokines, including IL-4 and IL-13, which contribute to the pathogenesis of CSU. Increased LPS uptake and reduced SCFAs contribute to abnormal differentiation of naive T cells, increasing their differentiation into Th2 and Th17 T cells, and decreasing the number of Th1 and Treg cells.^68^

High-fiber diets increase blood levels of propionic acid, blocking allergic inflammation via G-protein-coupled receptors (GPCR41), while butyrate and propionate inhibit IgE-dependent and non-dependent mast cell activation.^67^ On the other hand, the *Enterobacteriaceae* family (genera *Megamonas, Dialister*, and *Megasphaera*) recovered from patients with CU is one of the pro-inflammatory members of the intestinal microbiota of these patients, with a reduction in bacteria from the *Firmicutes* and *Bacteriodetes* strains that constitute 90% of the beneficial bacteria in the intestine being observed.^68^ Thus, intestinal dysbiosis is linked to CSU, as well as to other skin diseases such as psoriasis, atopic dermatitis, and acne.

Adult patients with CSU in endemic areas have more protozoa, showing a higher risk of seropositivity for toxocariasis and sensitization to *Anisakis simplex*, both *Ascarididae* helminths, compared to healthy controls. In children with CSU, infection by *Blastocystis hominis* is important.^68^ In addition to helminths, protozoa such as *Blastocystis hominis* and *Giardia lamblia* have the ability to produce high levels of specific IgE antibodies against host antigens, which can result in degranulation of mast cells in the human host.^68^ Furthermore, helminths disrupt the body's protective barriers, fostering a Th2 immune response and tissue repair, inhibiting Th1 differentiation, and encouraging the development of more Th2 cells. B cells support Th1 responses by producing IgG1 and IgG3, which can form immune complexes (CICs) with parasite antigens, activating anaphylatoxins (C3a and C5a) that act on mast cells, contributing to urticaria. These data demonstrate that the composition and alterations of the intestinal microbiome and parasites can have significant impacts on the pathogenesis of CSU.

### Pathogenesis of pruritus

Pruritus is the cardinal symptom of CSU. Although signs of excoriation are uncommon among patients with the disease, the pleasure of scratching the skin is extremely high among them, and much greater when compared to other pruritic dermatological diseases.^69^

Histamine released during mast cell degranulation and by basophils plays a prominent role in the pathogenesis of urticaria and its pruritus.^70^ Sensory nerve endings in the dermis have histaminergic pathways (H1 and H4 receptors), to which histamine binds and determines the perception of pruritus. However, with chronic disease, the Th2 immune pathway, supported by increased cytokines IL-4 and IL-5, activates mast cells, reducing their threshold for degranulation through the production of IgE and its binding to the FcɛRI receptor.^70^ Furthermore, both IL-31 and IL-33 are type 2 inflammatory cytokines and are crucial in the formation of hives and the induction of pruritus, since they bind to receptors in the non-histaminergic sensory pathways of the cutaneous nerves.^70^ Finally, proteases such as tryptase act on pruritogenic receptors in cutaneous sensory fibers (PAR1 and PAR2), serotonin (5-HT), in addition to the cytokines IL4, IL-13, and TSLP released by activated mast cells in CSU. Circulating basophils in the peripheral blood of patients with CSU synthesize IL-31 in response to IgE-dependent stimuli, as well as to IL-31 stimulation itself, which, in an autocrine manner, feeds back the release of IL-4 and IL-13.^70^ These cytokines also have receptors on sensory neural terminals in the non-histaminergic pathways. Therefore, pruritus in CSU is not solely histaminergic, as demonstrated by the ineffectiveness of second-generation H1 antihistamines in controlling it even at quadruple doses in a significant portion of patients, since the non-histaminergic pathway of pruritus is significantly important.^71^

## Biomarkers of Disease Activity and Therapeutic Response

Biomarkers, or “biological markers,” are objective and quantifiable characteristics of biological processes that refer to a broad subcategory of medical signs. These are objective indications of a medical condition, or a biological process, pathogenesis, or pharmacological response to a given therapeutic intervention, observed in patients. These findings can be accurately measured and are reproducible.^72^

Regarding CSU, there are no biomarkers related to diagnosis, except for clinical definition criteria.^73^, ^74^ Biomarkers in CSU can be divided into: (i) Clinical and serological biomarkers related to disease activity/clinical severity; (ii) Clinical and serological biomarkers of response to treatment. Such markers in CSU continue to be studied by several researchers, particularly cytokines/chemokines and substances expressed in the blood of patients^74^, ^75^ (Table 4).

**Table 4:** Biomarkers related to increased activity/severity of chronic spontaneous urticaria (CSU).^74^, ^75^, ^76^, ^77^

Clinical aspects	Hematological and/or serological aspects
• Older age at disease onset	• Low eosinophil count (<0.05 × 109/L)
• Female sex	• Low basophil count (<0.01 × 109/L)
• Presence of angioedema	• Low serum vitamin D levels
• Poor response to antihistamines	• Elevated CD2023c expression on basophils
• Positive autologous serum test	• Elevated eotaxin levels
• Hypersensitivity to nonsteroidal anti-inflammatory drugs (NSAIDs)	• Elevated VEGF levels
• Long-lasting CSU	• Elevated MMP-9 levels
	• Elevated IL-6 levels
	• Elevated IL-18 levels
	• Elevated IL-33 levels
	• Elevated C-reactive protein (CRP)
	• Elevated D-dimers (in half of patients with severe disease)
	• Elevated antithyroid antibodies (anti-TPO ≥ 34 kU/L)
	• Elevated mean platelet volume
	• Elevated erythrocyte sedimentation rate (ESR)
	• Positive basophil activation test (BAT)
	• Elevated serum adenosine levels
	• Elevated transglutaminase 2 activity

Regarding the therapeutic response, there are different markers that express a better or worse response^73^ to the main therapeutic agents proposed in the CSU treatment scale, by the International Consensus on Urticaria in 2022^74^: (i) Antihistamines [poor therapeutic response in patients with elevated UAS7 and concomitant CSU with induced CU, positivity of ASST and/or BAT (Basophil Activation Test), elevated CRP and/or ESR, and/or elevated serum D-dimer levels]; (ii) Omalizumab (late or poor response in the presence of signs of autoimmunity, such as positivity on ASST and BAT or ANA; adequate response in the absence of basophil-activating IgG autoantibodies, high expression of FcɛRI on basophils, and elevated serum total IgE levels; markers of omalizumab effectiveness are represented by increased basophil counts during treatment, decreased D-dimer and C-reactive protein levels, and reduced serum IL-31 levels); (iii) Cyclosporine: good response to treatment in the presence of signs of autoimmunity, such as positivity on BAT and/or ASST associated with low serum total IgE levels (<30–43 IU/mL)^76^; elevated d-dimer values correlate with a poor response to cyclosporine).^73^

## Stress and urticaria

CSU has a significant negative impact on quality of life (QoL), with approximately 40% of patients having a DLQI > 10 (severe or very severe impairment in QoL), resulting in considerable expenditures for the healthcare system (US$907 to US$2,084 annually per patient, especially due to the monetary costs of treatment).^76^, ^77^ In addition to the symptom burden that CU imposes on the patient and society, causing disturbances in sleep quality and productivity at work and school, the disease and its unpredictable course increase healthcare expenses for medications, outpatient and emergency room visits, hospitalizations, and laboratory tests, which are consequently associated with emotional imbalance, including mental conditions such as anxiety, depression, and stress, which can contribute to the persistence of the disease.^77^, ^78^

Any physical (e.g., illness, trauma, dehydration) or psychological (e.g., intense emotional stress) stimulus that imbalances homeostasis results in a systemic stress response.^77^ The brain is a receptor for stress stimuli and promoter of stress responses, transmitting stimuli to the periphery, such as the skin, through different mediators, either through a rapid and transient response mediated by the Sympathetic-Adrenomedullary (SAM) system or through a slow and transient/persistent response mediated by the Hypothalamic-Pituitary-Adrenal (HPA) axis.^77^

The acute stress response affects the immune system by increasing the secretion of IL-6, TNF-α, and IL-1β, mobilizing neutrophils through skeletal-muscle-derived neutrophil-attracting chemokines (CXCL-1) out of the bone marrow and temporarily directing these cells, lymphocytes, and monocytes from peripheral organs to the bone marrow through CXCR-4.^77^

Chronic stress can cause changes in innate and adaptive immunity through the actions of neuroendocrine mediators of the SAM and HPA axes.^77^ Stress causes the release of corticotropin-releasing hormone (CRH) by the hypothalamus, which activates the secretion of adrenocorticotropin (ACTH) by the anterior pituitary gland, which stimulates the adrenal cortex to release corticosteroids.^77^ As a negative feedback, elevated serum cortisol inhibits the secretion of CRH and ACTH.^77^ Chronic stress increases Th2 cell responses and reduces NK cell activity, also causing dysregulation of the HPA axis, leading to hypocortisolism and sustained hyperactivity of the sympathetic nervous system.^77^ Hypocortisolism is significantly associated with elevated levels of high-sensitivity CRP and IL-18, demonstrating the contribution of a vicious cycle of inflammation and hypocortisolism to the pathogenesis of CU.^77^

Furthermore, psychological stress can trigger the release of substance P by nerve endings in the skin, which activates the MRGPRX2 receptors on the mast cell membrane or Neurokinin-1 Receptors (NK1R), and together with the neural release of CGRP, constitute the link between stress and neurogenic inflammation, causing a bidirectional communication between mast cells and peripheral sensory neural endings. This is facilitated by the anatomical location of mast cells abundantly close to nerve endings, blood and lymphatic vessels, in the microvascular unit of the dermis, allowing a rapid response to environmental changes and participation in cutaneous inflammation.^77^ The increased expression of Corticotropin-releasing Hormone Receptor 1 (CRH-R1) in CU lesions also suggests a close connection between the skin and the HPA axis.

Some studies indicate that stressful events (loss of family members, financial difficulties, family conflicts, and work-related issues) precede CU exacerbations in a significant proportion of patients.^77^, ^78^ However, in light of current knowledge, it is important to exercise caution when interpreting the relationship between psychological stress and CSU.^76^, ^77^

## Comorbidities

CSU determines different areas of comorbidities, including sleep disorders (36.7%), depression (48.1%), anxiety (30.6%), mood disorders (29.4%), suicidal ideation (18.8%), and other psychiatric disorders (in 33% of patients), associated autoimmune diseases, atopic diseases, cardiovascular disorders, and, less frequently, an association with malignancies.^79^ Overall, approximately 30% of patients with CSU have at least one autoimmune disorder, while 2% may have one or more autoimmune diseases, with Hashimoto's thyroiditis and vitiligo constituting the most common coexisting diseases.^79^ Thyroid disease occurs in approximately 50% of patients with CSU, with a 5–7-fold increased risk of positive anti-TPO antibodies compared to controls. Other autoimmune diseases have a higher prevalence, depending on the population studied: pernicious anemia (>5%), vitiligo (>3%), insulin-dependent diabetes mellitus, rheumatoid arthritis, and celiac disease (>1% of patients, respectively).^79^ Approximately 80% of patients with CSU develop some autoimmune disease within ten years of their CSU diagnosis.^77^

Among children (<12 years) with CSU, ASST positivity, thyroid biological abnormalities, and the presence of antinuclear antibodies (ANA) were observed in 36.8%, 10.4%, and 6.4%, respectively, in addition to low serum vitamin D levels (69.1%) and psychiatric disorders (70.4%).^80^

Among adults with CSU, atopic comorbidities have been observed, including asthma (19.6%), allergic rhinitis (16.5%), atopic dermatitis (6.3%), and food allergy (8.2%), in data from the Scandinavian arm of the AWARE study.^81^ This pattern is similar to that observed in children with CSU, where atopic comorbidities were highly prevalent: atopic dermatitis (17.2%), allergic rhinitis (16%), asthma (13.2%), and food allergy (3.2%).^82^

Regarding malignancies, CU was associated with cancer in 0.007% of the population. It is resistant to H1 antihistamines, disappears after chemotherapy or tumor removal, can recur in tumor recurrence, and presents two to eight months before the diagnosis of malignancy.^79^ The most common malignancies are internal carcinomas (24% of which are papillary thyroid carcinomas), hematological malignancies, including non-Hodgkin's lymphoma, and stomach and liver cancer. However, it should be noted that although these cases are reported in the literature associated with CSU, the overall rates of cancer among patients with the disease are relatively low, so the international urticaria guidelines do not recommend screening for malignancies as a potential cause of the disease.^74^, ^79^

With the inflammatory skin condition and in persistent cases of CSU, an association is observed with metabolic syndrome, increased abdominal circumference, hyperlipidemia, hypertension, and cardiovascular disease.^79^ These conditions may be related to chronic inflammation and elevated TNF-α levels in these patients with CSU.^79^

## Urticaria and urticarial manifestations

CSU should be primarily differentiated from induced forms of chronic urticaria, such as pressure urticaria, symptomatic dermographism, cholinergic, solar, aquagenic, vibratory, and cold urticaria. These urticarias arise after exposure to an identifiable, usually external, triggering factor, unlike CSU; however, CSU can coexist with induced CU in approximately 30%–40% of patients, who may have more than one induced CU associated with CSU.^83^

Patients are advised to photograph their lesions approximately 48 hours before their appointment, as this can aid in visualizing the lesions and provide information about clinical characteristics, such as topography, shape, and size. This may indicate certain induced CU, persistent lesions, or residual brownish or purpuric hyperchromic lesions that suggest differential diagnoses such as urticarial vasculitis (UV).^84^

UV is the main differential diagnosis for CSU, and CSU and normocomplementemic UV can coexist in rare cases. UV should be suspected if at least one of the following characteristics occurs: hives lasting more than 24 hours in the same location, post-inflammatory purpura/hyperpigmentation, vesicles/blisters, residual desquamation, and associated systemic symptoms (arthralgia, fever, lymphadenopathy, etc.).^85^ On histopathology, the presence of leukocytoclasia and fibrin deposits constitutes the minimum criteria for the diagnosis of UV, and the presence of red blood cell extravasation is not exclusive.^85^ There is still debate as to whether CSU and normocomplementemic UV may constitute part of the same disease spectrum.^85^

Skin biopsy of the lesion is indicated in all cases where urticaria or angioedema are not characteristic, with typical symptoms and signs of urticaria/angioedema observed in CSU.^74^, ^86^

Situations have been proposed where skin biopsy is indicated in unusual situations in patients with CSU: absence of pruritus, presence of pain or burning prevailing over the pruritus symptom, presence of general symptoms (fever, arthralgia, lymphadenopathy, hepatomegaly, or splenomegaly), presence of a violaceous halo, purpura or residual hyperpigmentation, or laboratory findings with substantially elevated erythrocyte sedimentation rate and C-reactive protein values, alterations in protein electrophoresis (monoclonal gammopathy, in particular), exaggerated elevation of ferritin, and consumption of complement factors (C3, C4, CH50; hypocomplementemia). The authors also propose that the lack of response to appropriate treatment as recommended by the international urticaria guidelines would also constitute a warning sign for a skin biopsy.^86^, ^87^

An interesting semiotic resource for visualizing purpura in urticarial lesions is diascopy (with a glass slide or transparent plastic ruler), where compression fades the erythema of vasodilation and allows petechiae to persist, and dermoscopy, as demonstrated by Suh et al.,^88^ which can reveal linear vessels more commonly in CSU (86%), while the incidence of red-purplish dots or globules (90%) was more evident in the early stages of UV.

Other differential diagnoses for CSU include drug reactions, atypical bullous pemphigoid (urticarial), urticarial dermatitis, neutrophilic urticarial dermatosis (NUD/NUSI), autoinflammatory diseases with monogenic urticarial lesions, Schnitzler syndrome, and Adult-Onset Still's Disease (AOSD) (Table 5).^86^, ^87^

**Table 5:** Characteristics of chronic spontaneous urticaria and its main differential diagnoses.

	CSU	AIS	Schnitzler	NUD/NUSI	UV	UD	Drug	AIBD	Wells	AOSD
**Laboratory alterations**	None relevant	Elevated CRP, ESR, leukocytosis, neutrophilia, lymphopenia in some cases, elevated serum protein amyloid	Leukocytosis, neutrophilia, elevated CRP/ESR, IgM or IgA monoclonal gammopathy. Normal or elevated serum complement	Leukocytosis, neutrophilia, elevated CRP and ESR	May have complement consumption (↓C3 and/or ↓C4). In case of hypocomplementemia, it may present with cytopenias, ANA+, in addition to glomerulonephritis and COPD.	None relevant	Uncommon, possibly eosinophilia.	Eosinophilia, D-Dimer, and elevated total serum IgE in bullous pemphigoid. IgA antitransglutaminase antibodies in dermatitis herpetiformis.	Eosinophilia may occur.	Leukocytosis with neutrophilia, anemia, very high ferritin levels, elevated ceruloplasmin, elevated ESR and CRP during disease activity, as well as elevated AST and ALT and LDH, in addition to polyclonal gammopathy.
**Systemic manifestations**	Uncommon	Fever, arthralgias, arthritis, general malaise, visceromegaly	Arthralgia, osteosclerosis in the femur and tibia, then humerus, radius, ulna and fibula, lymphadenopathy, possibly visceromegaly	Fever, myalgia and fatigue, less frequently chest or abdominal pain.	In the hypocomplementemic form arthritis, uveitis, myalgias, serositis.	Usually absent or uncommon	Uncommon, except in serum sickness with urticarial manifestations, arthralgia, fever, and myalgia.	Uncommon	Rare.	Lymphadenopathy, sore throat, pretibial pain, visceromegaly, evening fever.
**Angioedema**	40‒60% of patients	Very rare	Rare	Absent	May be present	Absent	Uncommon, except in serum sickness	Absent.	Rare.	Absent in general.
**Residual hyperpigmentation/desquamation**	No	It may occur eventually	Possible	Absent	May be present along with purpura	Fine, light desquamation may occur.	Rare.	It may occur	It may occur	It may occur
**Associated symptoms**	Intense pruritus	No or minimal pruritus. Burning sensation may occur.	Pruritus is almost absent, or absent.	Absent or minimal pruritus.	Burning sensation and pain prevail over pruritus.	Intense pruritus	Pruritus	Intense pruritus	Burning sensation and pruritus.	Minimal or absent pruritus.
**Distribution of lesions**	Bilateral and asymmetric, in any area	Bilateral, usually symmetrical, in the trunk and extremities.	Asymmetrical, especially trunk and limbs.	Asymmetrical, trunk and limbs in particular.	Asymmetrical and all skin.	Bilateral, somewhat symmetrical, especially in the trunk and proximal limbs.	Bilateral and symmetrical, possibly in large folds	Generally symmetrical, varying with the bullous autoimmune disease in question.	Trunk and limbs.	Face, trunk and limbs.
**Types of lesions**	Hives and/or angioedema.	Nearly flat hives to infiltrated plaque lesions.	Urticated plaques and nearly flat urticarial lesions.	Macular erythema and/or palpable rash that resolves without sequelae and may be urticarial in nature.	Urticarial lesions that persist for more than 24 hours in the same location. Purpura and hyperpigmentation may be observed.	Pink, erythematous hives, slightly elevated, some with signs of mild lichenification. Excoriations are common.	Urticarial lesions sometimes associated with eczematous eruptions.	Urticarial lesions, possibly vesicles and/or erosions.	Hives with an arcuate or annular appearance. Occasional edematous-vesicular, papular lesions, and infiltrated plaques.	Urticarial lesions, slightly raised and persistent linear lesions, resembling dermographism.
**Duration of lesions**	Minutes or up to 24 hours	12 hours or a few days	>24 hours in the same place.	<24 hours	Two days to weeks in the same place	>24 hours and sometimes several days in the same place.	>24 hours	Several days or weeks in the same places.	>24 hours and sometimes several days in the same place.	>24 hours.
**Histopathology**	Nonspecific. Edema with Perivascular infiltrate of lymphocytes and polymorphonuclear cells. No epidermal changes.	There may be neutrophilic perivascular infiltrate and neutrophilic epidermotropism	There may be neutrophilic perivascular infiltrate and neutrophilic epidermotropism (epidermis, hair follicles, and eccrine glands)	Neutrophilic infiltration in the interstitium, between the collagen fibers, sometimes with a “single-file” appearance; without leukocytoclasis or vasculitis. No edema, and neutrophilic epidermotropism may be present.	Angiocentric infiltrate with or without interstitial spread. Leukocytoclasis and transmural polymorphonuclear cell infiltrate. Fibrin deposits in the blood vessel, red blood cell extravasation, and foci of thrombosis.	Mild spongiosis, perivascular infiltrate of lymphocytes and eosinophils, which may be in the superficial and middle dermis, is often mistaken for a drug reaction	Variable, with spongiosis, parakeratosis, vacuolar degeneration of the basal layer in the epidermis, keratinocyte apoptosis, perivascular inflammatory infiltrate of lymphocytes, eosinophils and/or neutrophils.	In lesions that are macroscopically free of vesicles or blisters in bullous pemphigoid, there may be eosinophilic spongiosis and eosinophils in the superficial dermis; in dermatitis herpetiformis, there may be neutrophils in the papillary dermis.	Discreet edema in the dermis with abundant eosinophils and “flame or blaze” figures, which are not pathognomonic of the disease.	Perivascular lymphomononuclear infiltrate with neutrophils and fewer eosinophils. Neutrophilic epidermotropism may be present.

CSU, Chronic Spontaneous Urticaria; AIS, Autoinflammatory Syndromes (monogenic ones include cryopyrinopathies related to NLRP3 mutations such as CINCA/NOMID, Mückle-Wells Syndrome, familial cold urticaria autoinflammatory syndrome, NLRP-12 mutations, possibly hyper-IgD syndrome and familial Mediterranean fever); NUD, Neutrophilic Urticated Dermatosis; NUSI, Neutrophilic Urticaria with Systemic Symptoms; UV, Urticarial Vasculitis; UD, Urticated Dermatitis; Drug, urticated drug reactions; AIBD, Autoimmune Bullous Diseases with Atypical Presentation (especially bullous pemphigoid and dermatitis herpetiformis); AOSD; Adult-Onset Still's Disease; COBPD, Chronic Obstructive Bronchopulmonary Disease; ANA, Antinuclear Factor; CRP, C-Reactive Protein; ESR, Erythrocyte Sedimentation Rate.

## Therapeutic Basis and Perspectives

The goal of CSU treatment is to achieve complete disease control, with the absence of symptoms and signs of the disease, as reflected by the application and collection of PROMs (Patient Report Outcome Measure: UCT, Urticaria Control Test, in a four-question format, and the seven-day Urticaria Activity Score, UAS7), with the goal of achieving a UCT = 16 and UAS7 = 0 in patients, in addition to a DLQI equal to zero, if possible. The achievement of these less subjective parameters demonstrates that the therapeutic intervention restored normality to the patient's life, aiming to interrupt the natural course of the disease, treating it until it disappears definitively.^74^, ^77^

In this therapeutic journey, various factors can trigger CSU exacerbations throughout treatment, even with completely asymptomatic periods, such as those reported in the Chronic Urticaria Registry (CURE)^89^: stress (13.9%), exposure to NSAIDs (6.7%), infections (5.5%; especially viral infections of the upper respiratory tract, including COVID-19 and its vaccines),^90^, ^91^, ^92^ and, rarely, foods such as milk, fish, seafood, nuts, peanuts, spices, fruits, chocolate, and alcohol.^77^, ^89^

The current international guidelines on urticaria provide a systemic treatment algorithm for CSU, which includes the use of daily second-generation antihistamines (anti-H1sg); Omalizumab (a humanized monoclonal anti-IgE IgG antibody) is prescribed monthly, and cyclosporine is prescribed daily, sometimes for long periods of years.^74^, ^77^

Old medications used to treat CSU before the advent of omalizumab, such as colchicine, dapsone, methotrexate, montelukast, hydroxychloroquine, H2-blocking antihistamines, and doxepin, have little scientific evidence due to the lack of randomized, double-blind, placebo-controlled studies with relevant case studies. Therefore, they are not included in the recommendations of the 2022 international urticaria guideline.^74^ However, in patients who do not respond to the measures recommended in the consensus, these medications can be used exceptionally.^93^

At least 25% to 50% of patients with CSU, even using quadruple doses of H1sg antihistamines, continue to have their disease without complete control, notably patients who have severe disease, concomitant CSU, and induced CU, together with elevated levels of d-dimers and serum CRP.^93^, ^94^, ^95^, ^96^, ^97^ Although H1sg antihistamines constitute the first line of treatment for CSU, a 2016 systematic review found that standard doses (from the “on-label” package insert) controlled 38.3% of patients, and 63.2% of patients required doses increased up to 4× the standard dose.^94^ There is evidence of effectiveness and, especially, safety in the use of up to four doses of H1s inhibitors as bilastine, cetirizine, levocetirizine, ebastine, fexofenadine, loratadine, desloratadine, mizolastine, and rupatadine^96^; approximately 32% of patients who did not respond to H1s inhibitors are also partial responders or non-responders to omalizumab, reflecting the heterogeneity of CSU pathogenesis among different patients.^93^, ^94^, ^95^, ^96^

H1sg inhibitors act on histamine receptors as inverse agonists, rendering them inactive when bound to them, reversing histamine-induced vasodilation and increased capillary permeability, reducing the edema that constitutes urticaria/angioedema.^96^ Furthermore, blocking the action of histamine on receptors in blood vessels and sensory nerve endings indirectly reduces allergic inflammation by decreasing the accumulation of inflammatory cells that migrate to the skin and suppress the immune response to antigens through its action on NFκB, as well as on calcium channels, endothelial cells, dendritic cells, and lymphocytes, also preventing the recruitment of eosinophils, basophils, neutrophils, and other immune cells from the blood to the skin.^96^

It should be noted that first-generation H1 antihistamines (H1fg – chlorpheniramine, diphenhydramine, hydroxyzine, promethazine, clemastine, doxepin, and cyproheptadine) were banned from the treatment of CSU due to their lower selectivity for H1 receptors, while also having affinity for muscarinic, serotonergic, and alpha-adrenergic receptors and cardiac potassium channels. These can cause side effects such as constipation, dry mouth, blurred vision, and can be potentially fatal. H1pg antihistamines are lipophilic drugs that cross the blood-cerebrospinal fluid barrier and, acting on the central nervous system, cause psychomotor changes, drowsiness, a comatose state, and decreased sleep quality (altering the REM sleep phase). These effects include side effects the day after nighttime use, decreased school and work performance, and an increased risk of drowsiness when driving.^96^

Due to their reduced passage through the blood-cerebrospinal fluid barrier, H1sg inhibitors are indicated for the treatment of CSU. However, elderly patients (≥65 years) may be particularly susceptible to some H1sg inhibitors, such as cetirizine and loratadine, when the label-recommended doses are exceeded.^11^, ^74^, ^96^

Omalizumab is indicated in addition to H1sg inhibitors when CSU is not controlled, as a second-line treatment, initially at the label-recommended doses of 300 mg subcutaneously every four weeks for at least six months.^74^ Its use is safe in pregnant women and is FDA category B in responding patients. A meta-analysis included randomized studies on the treatment of CSU/CIU, among patients older than 12 years of age, published between January 1, 2000, and July 31, 2021, recovering 854 articles, of which 14 met the inclusion criteria, comprising 577 participants using a placebo and H1 antihistamine and 1,209 participants with an active medication and an H1 antihistamine.^97^ It was observed that the effectiveness of omalizumab was dose-dependent, so that patients who are partial responders (to the on-label doses of 300 mg every four weeks) may benefit from increasing doses (450 mg or 600 mg every four weeks) or decreasing the interval to every two or three weeks between doses, which may be due to (i) the presence of higher serum levels of total IgE in these patients, since the most relevant pharmacological effect of omalizumab is to suppress the expression of IgE receptors. This action is due to the neutralization of free IgE in the blood, reducing it to almost zero, (ii) or the greater body weight of these patients.^97^

Compared to the immunosuppressants analyzed in this meta-analysis (including cyclosporine, methotrexate, azathioprine, and hydroxychloroquine), omalizumab was generally associated with greater reductions in DLQI and UAS7 values, while also demonstrating a lower incidence of adverse events.^97^ Cyclosporine, in turn, was effective in many patients and can be used in omalizumab-refractory patients or in clinical settings where economic conditions do not allow access to omalizumab. However, it has greater adverse effects when compared to omalizumab.^97^ Another meta-analysis involving 67 real-life studies with CSU patients treated with omalizumab demonstrated complete and partial response rates of 72% and 18%, respectively, with an average adverse event rate of 4%, such as headache, fatigue, and injection-site reactions.^98^

In patients with a complete response to omalizumab, anti-H1sg and omalizumab may be tapered every three months, discontinued after 6 to 12 months to determine if CSU remission has occurred.^77^ Alternative approaches to partial responses with omalizumab include using it in combination with immunosuppressive/immunomodulatory medications such as cyclosporine, dapsone, and colchicine, although the evidence for this use is very limited, or completely replacing it with cyclosporine, the third-line medication in the international urticaria guidelines.^11^, ^74^

Cyclosporine is a T-cell immunosuppressive agent (inhibiting the production of IL-2, IL-3, IL-4, and TNF-α), which also inhibits the release of mediators from mast cells and basophils, and has been used for over three decades in the treatment of CU.^11^, ^99^ Kulthanan et al.^99^ conducted a meta-analysis on the efficacy of cyclosporine in the treatment of CSU, involving 909 patients receiving doses between 1 and 5 mg/kg/day, having obtained a response rate to treatment after four weeks, at low doses (2-5 mg/kg/day) of 54% of patients, at eight weeks in 66% of them and at 12 weeks in 73% of patients, with patients presenting one or more adverse events with low doses (2-4 mg/kg/day) in 23% of them and 57% of those receiving moderate doses (4-5 mg/kg/day). Adverse events with the use of cyclosporine were dose-dependent, but the main ones were arterial hypertension and elevated serum creatinine in 6.2% with the use of low doses and 10.3% with moderate doses.^99^ Other events included gastrointestinal symptoms (nausea, vomiting, abdominal pain), headache, hirsutism, infections, and paresthesia, observed in 13.9% of patients with low doses and 46.2% of those with moderate doses, with studies with these doses conducted in less than 24 weeks and less than 16 weeks of cyclosporine use, respectively.^99^ It should be noted that prolonged use of cyclosporine, even at very low doses (< 2 mg/kg/day), has been shown over five to ten years to be associated with abnormal glomerular filtration rates and a higher incidence of infections and malignancies among patients.^99^

Within the current therapeutic approach proposed by the international urticaria guide published in 2022,^74^ there are, therefore, four medications with consensus regarding their effectiveness^53^: (i) Second-generation antihistamines, which target promoting inverse agonism of H1 receptors and blocking the undesirable effects of histamine; (ii) Omalizumab, which targets IgE sequestration and thus allows the suppression of its receptors on mast cells and basophils, dissociating the IgE bound to these cells in its receptor (FcɛRI) and blocking the activation of these cells; (iii) Cyclosporine, which generally inhibits T-cell activation and the release of histamine from mast cells and basophils, and (iv) Glucocorticoids, whose continuous use is contraindicated due to their adverse effects. However, they are useful in exacerbations of CSU when used in 7-day cycles per month, as they do not act directly on mast cells, but inhibit the recruitment of eosinophils from the bone marrow to the peripheral blood and tissues, in addition to blocking the influx of all blood cells into the interstitium of the dermis.^53^

New knowledge about the pathogenesis of CSU has led to the discovery of molecules (biological or small molecules) that can act not only on mast cells and basophils, but also on the predominantly Th2 inflammatory cell infiltrate, which depends on alarmins (TSLP, IL-33, IL-25) produced by epithelial and endothelial cells, which lead Innate Immune Lymphoid Cells type 2 (ILC2) to stimulate, with IL-13 and IL-5, the activation of LTh2, as well as on prostaglandin D2 receptors (CRTH2) that activate lymphocytes, mast cells and basophils.^53^ Receptors that stimulate the activation, differentiation, and survival of mast cells, such as c-Kit (CD117), are targeted by monoclonal antibodies such as barzolvolimab, blocking the action of its ligand, Stem Cell Factor (SCF). Other receptors that silence the mast cell and basophilic response, such as Siglec-8 (Sialic acid-binding immunoglobulin-like lectin-8) and Siglec-6, have been targeted by drugs with agonist function (anti-Siglec) to inhibit the function of these cells.^53^

There is a window of opportunity to act on this cascade of immune and inflammatory events, which also, to a lesser extent, involves LTh17 (for now, there are case reports with the use of secukinumab, an anti-IL-17, and tildrakizumab, an anti-IL-23).^100^, ^101^ Other biological medications undergoing phase II and III clinical trials, anti-IgEs, anti-TSLP, anti-IL-5 will soon have results published, in addition to dupilumab,^102^ which is awaiting registration by regulatory agencies such as the EMA (European Union) and FDA (USA) for the treatment of CSU, but are already approved in Japan, the United Arab Emirates and Brazil for the treatment of the disease. Small molecules for oral use, such as Bruton's tyrosine kinase inhibitors (an intracellular pathway that activates degranulation and cytokine synthesis in mast cells and basophils, as well as a pathway that stimulates antibody synthesis in B cells), such as remibrutinib, fenibrutinib, and risalbrutinib, have been studied in phase II and III studies with promising results.^53^

## Interleukin/cytokine receptor inhibitors or blockers

1) Dupilumab

Recent advances in understanding TH2 inflammation in CSU have opened new therapeutic horizons. Dupilumab is a human IgG4 monoclonal antibody that binds to IL-4Ra. This antibody inhibits IL-4 and IL-13-induced IL-4R signaling, reducing TH2 inflammation and potentially inhibiting IgE production in IL-4-treated B cells.^103^, ^104^ In a study that analyzed biopsies from patients with Chronic Spontaneous Urticaria (CSU), IL-4+ and IL-5+ cells were found in the lesional skin, with significant elevations compared to controls (patients without CSU). Furthermore, a marked increase in the number of IL-33+, IL-25+, and TSLP+ cells was observed in the dermis of lesional skin, compared to both non-lesional skin and the control group.^57^ The increased expression of Th2-initiating cytokines in the lesional skin of patients with CSU suggests that innate pathways may play a role in the mechanism of hives formation.^57^ Since Th2-initiating cytokines play a role in mast cell activation, inflammation, and vascular leakage, these findings may also have therapeutic implications for the use of dupilumab, for example.^57^

LIBERTY-CSU CUPID was a phase 3, multicenter, randomized, placebo-controlled, double-blind, 24-week trial.^105^ This trial included two independent studies, CUPID A and CUPID B, with different populations and inadequate response to different prior therapies of the same duration. Study CUPID A included patients who had never used omalizumab, and CUPID B included patients intolerant to omalizumab or with an incomplete response.^105^ In CUPID A, the combination of dupilumab with second-generation antihistamines provided significant and clinically relevant improvements at week 24, including pruritus, urticaria, and urticaria activity, compared to antihistamines alone.^105^ In CUPID B, patients were defined as incomplete responders after three months of treatment with omalizumab, and in this group, there was a statistically significant improvement in pruritus and urticaria assessments after the use of dupilumab.^105^, ^106^ In December 2024, dupilumab was approved by ANVISA in Brazil for the treatment of CSU unresponsive to antihistamines in adolescents over 12 years of age and adults.

2) Anti-IL-5

In CSU, IL-5 is responsible for attracting eosinophils to the skin, in addition to promoting their survival and proliferation.^107^ One study demonstrated that patients with CSU have higher serum levels of IL-5 receptors than controls.^108^ Another study detected higher concentrations of eosinophils in both lesional and non-lesional skin of individuals with CSU compared to controls.^109^

There are some case reports in which mepolizumab, an anti-IL5 monoclonal antibody, was successfully used for the treatment of CSU.^110^ An open-label, exploratory, phase 1 study is currently being conducted to evaluate mepolizumab for the treatment of CSU.^111^

Benralizumab, an IL-5 receptor inhibitor (anti-IL-5Rα), underwent a phase 1 study with promising results in CSU.^112^ However, these results were not corroborated in the randomized, placebo-controlled clinical trial. Therefore, its development program for CSU was discontinued.^113^

3) Anti-IL-17 – Secukinumab and Anti-IL-23 – Tildrakizumab

In a study of patients with CU, the levels of circulating inflammatory cytokines such as IL-6, TNF-α, and IL-12 were evaluated.^114^ These were found at elevated levels in patients with chronic urticaria, in association with increased secretion of IL-2 and IL-17 after T-cell stimulation.^60^, ^114^ In another study, serum levels of IL-17, TNF-α, and IL-23 were evaluated in patients with CSU, in relation to the values of the urticaria activity scale, positive autoserum test in patients, and prick test results.^115^ The concentration of these cytokines was significantly higher in patients with CSU compared to controls in all of these studies.^60^, ^114^, ^115^

In a recent study, patients with severe CSU resistant to conventional treatment were treated off-label with anti-IL-17A (secukinumab – 150 mg weekly for four weeks and then every two weeks).^116^ The improvement in disease activity (UAS7) was 55% at 30 days and 82% at three months. Discontinuation of treatment after six to eight months resulted in CSU relapse, indicating that anti-IL-17 would be necessary for a longer period.^116^

A case series reported the use of tildrakizumab, an anti-IL-23 agent, in three patients with high disease activity refractory to treatment with quadrupled doses of second-generation H1 antihistamines and omalizumab.^117^ These patients received three doses of tildrakizumab 100 mg (at weeks 0, 4, and 12, the dose and interval used as licensed for psoriasis) as an off-label treatment, while maintaining antihistamines at quadruple doses. The treatment was well tolerated and led to significant improvements in disease activity and control in all patients after 12 weeks, with reductions in UAS7 and UCT scores and improvements in quality of life.^117^ One patient achieved remission, another maintained adequate control with antihistamines, and one relapsed after stopping the drug.^117^

4) Anti-thymic stromal lymphopoietin (TSLP): Tezepelumab

Thymic stromal lymphopoietin (TSLP) is an epithelial-derived cytokine (alarmin) produced in response to environmental and pro-inflammatory stimuli. It is associated with the regulation of type 2 immunity, acting on dendritic cells, T and B cells, and innate immune lymphoid cells (ILC2).^10^ TSLP expression is higher in the airways of patients with asthma, and its levels correlate with the expression of Th2 cytokines and chemokines and disease severity.^10^, ^118^

Tezepelumab (AMG 157/MEDI9929) is a human IgG1 monoclonal antibody undergoing investigation that binds to TSLP, preventing its interaction with the TSLP receptor complex.^118^ In a phase 2b study registered in Clinical Trials (INCEPTION study) with a 32-week observation period, comparing the effect of Tezepelumab (210 mg every four weeks or 420 mg every two weeks) versus omalizumab (300 mg every four weeks) or placebo, in patients refractory to optimized treatment with quadrupled doses of H1 antihistamines, a sustained treatment effect was observed in the Tezepelumab arms, not achieved with omalizumab or placebo.^119^ Improvements in UAS7 values with the drug occurred with continued reductions in IL-5 and IL-13, regardless of changes in IgE levels.^119^ In patients who never used anti-IgE, Tezepelumab treatment led to reductions in CSU activity and biomarkers after treatment discontinuation, suggesting a sustained effect of TSLP blocking after treatment cessation.^10^, ^119^

### Bruton's Tyrosine Kinase Inhibitors (BTK Inhibitors)

1) Remibrutinib

Remibrutinib (LOU064), an oral, covalent (stable-binding) BTK inhibitor, exhibits high selectivity and potency for this intracellular signaling pathway molecule in mast cells, basophils, and B cells. The high selectivity and tolerability of remibrutinib are likely due to its ability to bind to an inactive conformation of BTK.^120^

In a phase I study, remibrutinib was also effective in inhibiting basophil activation.^121^ In a phase 2b clinical trial, the efficacy and safety of oral remibrutinib at various doses were demonstrated in patients with CSU inadequately controlled with second-generation antihistamines.^122^ All doses of remibrutinib (10 mg/day, 35 mg/day, 100 mg/day, 10 mg twice daily, 25 mg twice daily, and 100 mg twice daily) significantly improved the signs and symptoms of CSU compared to placebo, with rapid clinical improvement observed in the first week (improvement of pruritus and hives) and maintained until week 12.^122^ Greater overall efficacy was observed with remibrutinib 25 mg twice daily compared with placebo.^122^

Prior treatment with anti-IgE therapy did not affect the change in UAS7 score from baseline to week 12, as no relevant difference was observed between anti-IgE-experienced and naive patients in either the remibrutinib (any dose) or placebo arms.^122^ Remibrutinib demonstrated a favorable safety profile at all tested doses.^122^ Most Adverse Events (AEs) were mild or moderate, and the proportion of patients with at least one AE was similar across remibrutinib doses, indicating no dose-dependent pattern.^122^ Similarly, the extension study of remibrutinib, observing patients who had UAS7 ≥ 16 in the previous 16-week study, remained in this study with remibrutinib at a dose of 100 mg twice daily for 52 weeks, in order to evaluate adverse events, the three most common of which were infections (30.9%) in the skin and subcutaneous tissue (26.8%) and gastrointestinal disorders (16.5%). UAS7 = 0 was achieved by 28.2% at week 4 and 55.8% at week 52, and UAS7 ≤ 6 by 52.5% at week 4 and 68.0% at week 52, demonstrating the efficacy and safety of this medication.^123^

2) Fenebrutinib

Fenebrutinib is an oral, highly selective, and reversible BTK inhibitor that blocks IgE-mediated histamine release from mast cells *in vitro*. In a phase 1 study, fenebrutinib inhibited IgE-mediated basophil activation in healthy volunteers. Thus, BTK inhibition by fenebrutinib may also interrupt the production of autoantibodies that activate FcεRI in CSU. A phase 2b, multicenter, randomized, double-blind, placebo-controlled study involving 134 participants in 57 days of treatment, distributed in a pilot cohort (cohort 1, dose of 200 mg, twice a day) and a variable-dose cohort (cohort 2, subdivided into doses of 50 mg daily, 150 mg daily and 200 mg twice a day), observed the efficacy and safety of fenebrutinib, compared to placebo, in adult patients with CSU for more than six months, symptomatic despite treatment with H1 antihistamines at an optimized dose (up to four times the approved dose in the package insert).^124^ The rapid onset of efficacy suggests that the main mechanism of action of the drug in CSU is inhibition of the FcεRI signal via inhibition of BTK in mast cells and basophils.^125^ Fenebrutinib controlled CSU in patients with type IIb autoimmunity at all tested doses.^124^, ^125^

However, at lower doses, patients with type IIb autoimmunity experienced greater benefit than patients without type IIb-associated markers.^125^ Patients with type IIb autoimmunity may be more sensitive to BTK inhibition, while patients with type I autoallergy may derive additional clinical benefit from higher levels of pathway inhibition to achieve maximal efficacy.^125^ The primary endpoint of achieving a UAS7 ≤ 7 was achieved with the 200 mg twice daily and 150 mg daily doses, but not with the 50 mg daily dose of fenebrutinibe. Asymptomatic and reversible grade 2 and 3 transaminase elevations occurred with the 150 mg daily and 200 mg twice daily doses (two participants in each group).^125^

### MGPRX2 Receptor Inhibitor

In addition to the high-affinity IgE receptor (FcεRI), mast cells express numerous G-protein-coupled receptors (GPCRs), which can be activated by neuropeptides, major basic protein (MBP), and eosinophil peroxidase (EPO), among others, culminating in mast cell degranulation.^126^ Mas-related G-protein-coupled receptor X2 (MRGPRX2) is a GPCR expressed with some selectivity by mast cells and can be present both in the plasma membrane and intracellularly.^126^ MRGPRX2 expression is increased in the skin of individuals with CSU when compared with healthy controls.^127^ Fujisawa et al. ^127^ also observed eosinophil infiltration in the lesional skin in seven out of nine individuals with CSU. The authors also demonstrated that proteins originating from eosinophils (MBP and EP) are capable of inducing histamine release; this effect is inhibited in cells in which MRGPRX2 is silenced.^127^ An oral MRGPRX2 inhibitor (EP262) is currently recruiting for a phase 2 clinical trial for CSU, a study called CALM-CSU.^128^

### Tyrosine kinase receptor (KIT) inhibitors (c-KIT ligand inhibitors)

KITs (CD117) are transmembrane receptors for Stem Cell Factor (SCF) and are present on progenitor cell lines.^129^ In the case of mast cells, even mature cells continue to express KIT at high levels.^129^ When activated by SCF, KIT promotes cell survival, migration, and the secretion of several inflammatory mediators.^129^ In mast cells, KIT activation by SCF induces degranulation.^129^

Barzolvolimab is a humanized monoclonal antibody that binds to the KIT extracellular domain on mast cells, preventing SCF binding and subsequent mast cell activation.^130^ In a randomized phase 2 clinical trial for the treatment of CSU, barzolvolimab demonstrated a significant improvement in the UAS7 score compared to the placebo group, over a 12-week follow-up.^131^ No major adverse effects were observed in this same study.^131^ In Chronic inducible urticaria (CIndU), specifically symptomatic dermographism and cold urticaria, there was complete resolution of the condition in 95% of patients after a single infusion of barzolvolimab, over a 12-week follow-up.^132^ Furthermore, there was a significant reduction in the number of mast cells in the patients' skin, as well as a reduction in tryptase levels and an increase in soluble SCF after treatment.^132^ Barzolvolimab is currently being investigated in phase 3 clinical trials for CSU and is considered a promising treatment for CSU (EMBARQ-CSU2 study).^133^

Briquilimab is another anti-KIT monoclonal antibody that is being evaluated for the treatment of CSU, but is still recruiting participants.^134^

### Anti-Siglecs Medications

Siglecs, short for sialic-acid-binding immunoglobulin-like lectins, are a family of receptors expressed on mast cells, eosinophils, and basophils.^135^ They have an inhibitory function, acting as immune system checkpoints.^135^ Siglec-6 and -8 have been explored as therapeutic targets in allergic diseases.^135^ Siglec-8 induces eosinophil apoptosis and inhibits mast cell activation; siglec-6 appears to promote even more potent inhibitory signals in mast cells.^135^, ^136^

Lirentelimab (AK002), an anti-Siglec-8 monoclonal antibody, has been evaluated for the treatment of CSU. Despite showing promising results in an initial proof-of-concept study, the subsequent phase 2 clinical trial did not meet the primary endpoint and was terminated early by the manufacturer.^137^ A humanized IgG1 anti-Siglec-6 monoclonal antibody (AK006) is currently being investigated in clinical trials for the treatment of CSU.^138^

## Conclusions

Urticaria, and CSU in particular, is a disease with rapidly expanding knowledge regarding its pathophysiological aspects, which are the basis for the development of new drugs for its management.

Many genetic aspects that may predispose individuals to the disease, the exogenous factors that play a variable and individual role, the recognition of specific endotypes (autoallergic and autoimmune), the central role of mast cell degranulation, the important role of different cells and mediators, comprising diverse mechanisms in the pathogenesis, and biomarkers have been established; however, there are still gaps to be addressed.

Studies on new drugs for the treatment of CSU reflect the multiplicity of pathogenic pathways. Once clinical trials demonstrate efficacy and safety, as has already been the case with omalizumab and others, regimens with immunobiologicals and/or small molecules will likely be considered alternatives to current treatments, especially in cases of resistance to conventional management.

The fight for a better quality of life for patients with CSU is constant, and science is on our side, reminding us that promising therapies must be accompanied by concerns about accessibility, correct indication, and clinical monitoring.

## Editor

Ana Maria Roselino.

## Financial support

None declared.

## Research data availability

The entire dataset supporting the results of this study was published in this article.

## Authors’ contributions

Hélio Amante Miot: Study design, drafting and editing of the manuscript, and approval of the final version of the manuscript.

Roberta Fachini Jardim Criado: Study design, drafting and editing of the manuscript, and approval of the final version of the manuscript.

Paulo Ricardo Criado: Study design, drafting and editing of the manuscript, and approval of the final version of the manuscript.

Helena Zenedin Marchioro: Study design, drafting and editing of the manuscript, and approval of the final version of the manuscript.

Renan Rangel Bonamigo: Study design, drafting and editing of the manuscript, and approval of the final version of the manuscript.

Beatrice Martinez Zugaib Abdalla: Study design, drafting and editing of the manuscript, and approval of the final version of the manuscript.

## Conflicts of interest

Paulo Criado: Advisory board ‒ Pfizer, Galderma, Takeda, Hypera, Novartis, Sanofi; Clinical research - Pfizer, Novartis, Sanofi, Amgen and Lilly; Lecturer: Pfizer, Abbvie, Sanofi-Genzyme, Hypera, Takeda, Novartis.

Roberta Fachini Jardim Criado: Advisory board ‒ Pfizer, Takeda, Hypera, Novartis, Sanofi; Clinical research - Pfizer, Novartis, Sanofi and Lilly; Lecturer: Pfizer, Abbvie, Sanofi-Genzyme, Hypera, Takeda, Novartis.

Hélio Miot: Advisory Board – Johnson & Johnson, L’Oréal, Theraskin, Sanofi e Pfizer; clinical research Abbvie, Galderma and Merz.

Beatrice Martinez Zugaib Abdalla – No conflicts of interest declared.

Helena Marchioro – Advisory Board – Novartis and UCB Pharm; Lecturer: Johnson & Johnson, Novartis and UCB Pharma.

Renan Bonamigo – No conflicts of interest declared.
